# Identification of Recessive Lethal Alleles in the Diploid Genome of a Candida albicans Laboratory Strain Unveils a Potential Role of Repetitive Sequences in Buffering Their Deleterious Impact

**DOI:** 10.1128/mSphere.00709-18

**Published:** 2019-02-13

**Authors:** Timea Marton, Adeline Feri, Pierre-Henri Commere, Corinne Maufrais, Christophe d’Enfert, Melanie Legrand

**Affiliations:** a Institut Pasteur, INRA, Unité Biologie et Pathogénicité Fongiques, Paris, France; b Université Paris Diderot, Sorbonne Paris Cité, Cellule Pasteur, Paris, France; c Institut Pasteur, Unité de Technologie et de Service Cytométrie et Biomarqueurs, Plate-Forme Cytométrie, Paris, France; d Centre de Bioinformatique, Biostatistique et Biologie Intégrative (C3BI), USR 3756 IP CNRS, Institut Pasteur, Paris, France; Carnegie Mellon University

**Keywords:** *Candida albicans*, homologous recombination, loss of heterozygosity, major repeat sequences, recessive lethal alleles

## Abstract

The heterozygous diploid genome of Candida albicans is highly plastic, with frequent loss of heterozygosity (LOH) events. In the SC5314 laboratory strain, while LOH events are ubiquitous, a chromosome homozygosis bias is observed for certain chromosomes, whereby only one of the two homologs can occur in the homozygous state. This suggests the occurrence of recessive lethal allele(s) (RLA) preventing large-scale LOH events on these chromosomes from being stably maintained. To verify the presence of an RLA on chromosome 7 (Chr7), we utilized a system that allows (i) DNA double-strand break (DSB) induction on Chr7 by the I-*Sce*I endonuclease and (ii) detection of the resulting long-range homozygosis. I-*Sce*I successfully induced a DNA DSB on both Chr7 homologs, generally repaired by gene conversion. Notably, cells homozygous for the right arm of Chr7B were not recovered, confirming the presence of RLA(s) in this region. Genome data mining for RLA candidates identified a premature nonsense-generating single nucleotide polymorphism (SNP) within the HapB allele of C7_03400c whose Saccharomyces cerevisiae ortholog encodes the essential Mtr4 RNA helicase. Complementation with a wild-type copy of *MTR4* rescued cells homozygous for the right arm of Chr7B, demonstrating that the mtr4^K880*^ RLA is responsible for the Chr7 homozygosis bias in strain SC5314. Furthermore, we observed that the major repeat sequences (MRS) on Chr7 acted as hot spots for interhomolog recombination. Such recombination events provide C. albicans with increased opportunities to survive DNA DSBs whose repair can lead to homozygosis of recessive lethal or deleterious alleles. This might explain the maintenance of MRS in this species.

**IMPORTANCE** Candida albicans is a major fungal pathogen, whose mode of reproduction is mainly clonal. Its genome is highly tolerant to rearrangements, in particular loss of heterozygosity events, known to unmask recessive lethal and deleterious alleles in heterozygous diploid organisms such as C. albicans. By combining a site-specific DSB-inducing system and mining genome sequencing data of 182 C. albicans isolates, we were able to ascribe the chromosome 7 homozygosis bias of the C. albicans laboratory strain SC5314 to an heterozygous SNP introducing a premature STOP codon in the *MTR4* gene. We have also proposed genome-wide candidates for new recessive lethal alleles. We additionally observed that the major repeat sequences (MRS) on chromosome 7 acted as hot spots for interhomolog recombination. Maintaining MRS in C. albicans could favor haplotype exchange, of vital importance to LOH events, leading to homozygosis of recessive lethal or deleterious alleles that inevitably accumulate upon clonality.

## Abstract (precis)

Candida albicans is a major fungal pathogen, whose mode of reproduction is mainly clonal. Its genome is highly tolerant to rearrangements, in particular loss of heterozygosity events, known to unmask recessive lethal and deleterious alleles in heterozygous diploid organisms such as C. albicans. By combining a site-specific DSB-inducing system and mining genome sequencing data of 182 C. albicans isolates, we were able to ascribe the chromosome 7 homozygosis bias of the C. albicans laboratory strain SC5314 to an heterozygous SNP introducing a premature STOP codon in the *MTR4* gene. We have also proposed genome-wide candidates for new recessive lethal alleles. We additionally observed that the major repeat sequences (MRS) on chromosome 7 acted as hot spots for interhomolog recombination. Maintaining MRS in C. albicans could favor haplotype exchange, of vital importance to LOH events, leading to homozygosis of recessive lethal or deleterious alleles that inevitably accumulate upon clonality.

## INTRODUCTION

In diploid genomes, new mutations are heterozygous, and their effect is generally masked by the presence of the ancestral allele. As claimed in Haldane’s sieve, only mutations that confer a fitness advantage as heterozygotes can invade the population. Although true, it does not make specific prediction about the fitness of the mutant homozygotes. Recent studies of Saccharomyces cerevisiae have observed maintenance of genetic variation due to heterozygote advantage, as a result of overdominance of mutated alleles (1). In addition, Gerstein and colleagues used the model organism S. cerevisiae to show that recessive beneficial mutations can avoid Haldane’s sieve in clonal organisms, through rapid loss of heterozygosity (LOH), and thus contribute to rapid evolutionary adaptation (1). Similarly, in Candida albicans, mutations followed by genomic rearrangements such as LOH events and isochromosome formation have been associated with the acquisition of antifungal resistance (2, 3), bringing forth the idea that mechanisms favoring genome plasticity could contribute to C. albicans fitness within the host and upon exposure to antifungal agents. C. albicans is a frequent human commensal yeast responsible for both mucosal fungal infections and the majority of life-threatening nosocomial fungal infections (4). Its diploid genome displays a high degree of plasticity that includes, in particular, LOH events. Despite frequent LOH, overall heterozygosity is maintained in the C. albicans population, as illustrated by various studies that highlighted the elevated levels of natural heterozygosity, with a heterozygous position every ∼200 to 250 bp on average (5 – 7). Furthermore, several studies revealed that genome heterozygosity showed a significant correlation with higher growth rates (6, 8, 9).

Essentially, mutations can be categorized as beneficial, harmful, or neutral and can be differently assigned depending on the organism’s environment. Because the mode of reproduction of C. albicans is mainly clonal, and therefore mimics inbreeding in higher eukaryotes, an increased number of recessive lethal alleles (RLA) in the C. albicans genome is expected compared to other eukaryotes that undergo true sexual reproduction. Various types of mutations can impact the functionality of alleles and render them inactive; however, mutations introducing premature STOP codons would convey the most obvious effect. Within the C. albicans laboratory strain SC5314, Muzzey et al. (10) reported almost 200 genes for which one of the alleles contains a single nucleotide polymorphism (SNP) that introduces a premature STOP codon. Functional differences have already been reported for the two alleles of a heterozygous gene, and in all instances, the effect of the recessive mutation was visible only upon homozygosis toward the mutated allele (*HIS4* [11], *MBP1* [12], *GPI16/MRF2* [13]). Moreover, SNPs in promoter regions have been shown to alter expression regulation of two alleles (14). Of interest, LOH is pervasive in C. albicans isolates, as homozygous regions can be found in all sequenced isolates and affect all of the chromosomes. These LOH events vary in size: they can be limited to a single chromosomal region, affect an entire chromosomal arm, or even cover the entire chromosome (6, 7, 9).

Recently, a combination of molecular tools has been developed to study genome dynamics in C. albicans. First, an LOH reporter system takes advantage of fluorescent markers at an artificial heterozygous locus containing the BFP and GFP genes (15). Consequently, the appearance of spontaneous LOH events for the given locus can be monitored by the fluorescent status of cells using flow cytometry (15). Second, LOH events are often a result of DNA double-strand breaks (DSB) (16) resolved by means of various DNA repair mechanisms which can either be independent or dependent of homologous recombination. Feri et al. (13) developed an inducible, locus-specific DNA DSB system that uses the I-*Sce*I meganuclease from S. cerevisiae. When coupled to the BFP/GFP LOH reporter system, this system can be used to study the consequence of a targeted DNA DSB on the appearance of LOH events. Indeed, Feri et al. (13) have shown that I-*Sce*I-induced DNA breaks are predominantly repaired by gene conversion resulting in limited LOH. Nevertheless, various patterns of long-range LOH can also be obtained. Of note, the engineered system, alongside sequence resources, helped identify a RLA on Chr4B of C. albicans strain SC5314 (13). This RLA is the consequence of a nonsense mutation in the *GPI16* gene that encodes an essential component of the glycosylphosphatidylinositol (GPI) anchor biosynthetic machinery and explains why Chr4B is never observed in the homozygous state in C. albicans strains SC5314. Notably, this RLA appeared unique to strain SC5314 (13).

Although LOH can be observed on all eight chromosome pairs, prior studies conducting haplotype characterization of (i) progeny from the parasexual life cycle (17), (ii) homozygous diploid isolates derived from *RAD*52 double knockout mutants (18), and (iii) haploid strains of C. albicans (8) showed a chromosome homozygosis bias in the C. albicans laboratory strain SC5314. This suggests that mutations, potentially RLAs, could apply constraints on the directionality of LOH events. Indeed, the homozygosis state of some chromosomes was observed only for a given homolog while recurrently absent for the other homolog. This is the case for chromosomes 1, 4, 6, as well as chromosome 7 (Chr7) for which homozygosis of haplotype B (HapB) is never observed, while haplotype A homozygosis is, suggesting the presence of RLAs on Chr7 HapB (Chr7B).

In this study, we aimed to identify the RLA(s) on Chr7B using an approach similar to that developed by Feri et al. (13) when searching for RLAs on Chr4B. This approach also allowed addressing the role that repetitive sequences, such as the major repeat sequences (MRS), might play on the overall genome dynamics of C. albicans. MRS are unique to C. albicans and Candida dubliniensis, and are found throughout their genomes. MRS are composed of three subregions: RB2 which contains the *FRG8* gene, the RPS region which varies in repeat numbers (and thus, in size), and the HOK region. C. albicans possesses eight MRS, one on each chromosome with the exceptions of Chr7 where the presence of one MRS on each arm is observed and of Chr3 where an incomplete MRS is located (19). MRS expansion and contraction have previously been shown to be involved in chromosome loss where the chromosome copy containing the shorter MRS region is spontaneously lost (20). Furthermore, MRS have also been shown to be involved in chromosome translocation (19), when two different chromosomes exchange large regions of an arm. Results presented below confirm that homozygosis of Chr7B is not recovered and that this is the consequence of a premature STOP codon in the Chr7B-borne allele of the RNA helicase-encoding gene *MTR4*. Furthermore, we highlight that repeat regions such as MRS are hot spots for interhomolog recombination upon DNA repair and play a role in LOH dynamics in C. albicans.

## RESULTS

### Strain engineering to promote and detect long-range LOH on Chr7.

**FIG 1:**
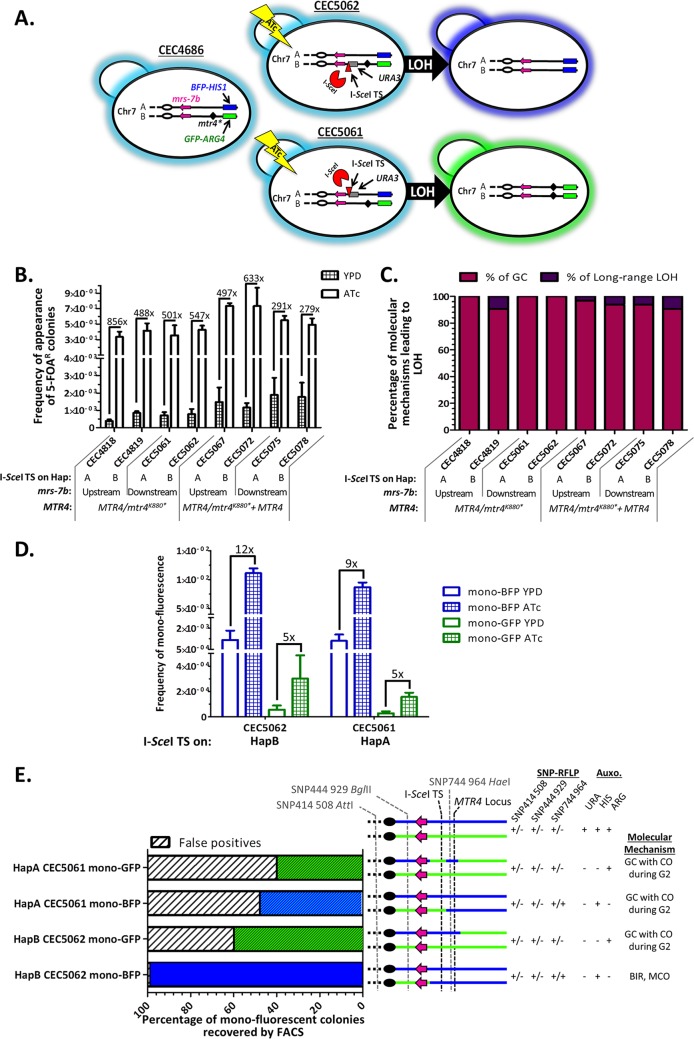
Coupling of a DNA double-strand-break-inducing system and a FACS-optimized LOH reporter system on Chr7. (A) Illustration of strains bearing the BFP/GFP LOH reporter system on the right arm of Chr7 associated with an I-*Sce*I-inducible DNA DSB system on Chr7B (CEC5062) or Chr7A (CEC5061). Upon addition of ATc, the mega-endonuclease I-*Sce*I is expressed and will generate a DNA DSB at its target sequence (I-S*ce*I TS linked to the *URA3* marker) on Chr7. Theoretically, when the DNA DSB is repaired by break-induced replication or mitotic crossover, doubly fluorescent cells harboring the I-*Sce*I-TS on BFP-bearing Chr7A will become mono-GFP, while doubly fluorescent cells harboring the I-*Sce*I-TS on GFP-bearing Chr7B will become mono-BFP. The centromere (oval), MRS (pink arrow), and candidate RLA (diamond) on Chr7 are shown. (B) Average frequencies of 5-FOA^R^ colonies obtained after 8 h of I-*Sce*I overexpression and recovery from three independent experiments (*n* = 3) (plus standard deviations [S.D.] [error bars]) alongside fold changes observed between YPD and ATc conditions. (C) Percentage of molecular mechanisms leading to LOH among 5-FOA^R^ colonies in induced condition (*n* = 32). (D) Histogram representing average frequency (*n* = 6) (plus S.D.) of appearance of monofluorescent cells in the presence (ATc) and absence (YPD) of expression of I-*Sce*I and hence induced DNA DSBs on Chr7B (CEC5062) or Chr7A (CEC5061). Fold changes between uninduced and induced conditions for each monofluorescent population are indicated. (E) Characterization of monofluorescence-sorted individuals (*n* = 32) from induced CEC5062 (I-*Sce*I TS on HapB) and CEC5061 (I-*Sce*I TS on HapA) strains. A subset of sorted individuals was characterized for fluorescence and auxotrophy status in addition to SNP-RFLP for haplotype appointment in order to profile homozygosis of the right arm of Chr7 and determine the molecular mechanism used for I-*Sce*I-induced DNA DSB repair. In the stacked histogram, white and black hatched portions represent missorted cells (false-positive results), while green and blue portions represent the proportions of properly FACS-sorted monofluorescent individuals. The presence of stripes on the green or blue bars indicate that those individuals are not fully homozygous for one given haplotype from the I-*Sce*I TS to the BFP/GFP LOH reporter system on the right arm of Chr7. Abbreviations of molecular mechanisms are as follows; gene conversion (GC), break-induced replication (BIR), mitotic crossover (MCO), gene conversion with crossover (GC with CO).

Genome analysis revealed that the left arm of Chr7 carries only 9 heterozygous SNPs in 3 ORFs, while the right arm of Chr7 carries 784 heterozygous SNPs in 105 ORFs. Because RLAs are more likely to be found in heterozygous regions, we focused on the right arm of Chr7 to understand Chr7 homozygosis bias. To efficiently screen for the presence of RLAs on Chr7 right arm, we engineered strains carrying an artificial heterozygous BFP/GFP LOH reporter system (15) associated with an I-*Sce*I DNA DSB-inducing system (13) (Fig. 1A). Because we chose to insert the I-*Sce*I target site (TS) associated with the auxotrophic marker *URA3* conferring uridine prototrophy in the most *mrs-7b* proximal, gene-free region found on the right arm of Chr7, this setup allows rendering a maximum number of alleles homozygous on this arm while avoiding *mrs-7b*. Integration of the *URA3*-I-*Sce*I TS cassette can occur on either Chr7 homologs (Chr7A or Chr7B); thus, transformants were screened by SNP-RFLP to identify which Chr7 haplotype was targeted (see Fig. S1 in the supplemental material). Using the heterozygous SNP at position 727,328, we showed that 28/51 C. albicans transformants had integrated the I-*Sce*I TS on Chr7A (55%) and 23/51 on Chr7B (45%), demonstrating the absence of integration bias for this locus. Two independent transformants that had integrated the I-*Sce*I TS on Chr7 HapA (CEC5061) or HapB (CEC5062), were selected and used in subsequent analysis (Fig. 1A).

10.1128/mSphere.00709-18.2 FIG S1 Overview of Chr7 in engineered strains throughout this study. Schematic localization of the I-*Sce*I target sequences relative to the MRS, the BFP/GFP LOH reporter system, and the *MTR4*/*mtr*4^K880*^ locus. Sites used for SNP-RFLP and associated restriction enzymes are shown, where a rhombus identifies the enzyme-sensitive allele. Download FIG S1, TIF file, 0.1 MB. Copyright © 2019 Marton et al. 2019 Marton et al. This content is distributed under the terms of the Creative Commons Attribution 4.0 International license.

Strains CEC5061 and CEC5062 underwent preliminary characterization regarding their fluorescence status, as well as their growth rate to ensure that the successive transformation steps did not significantly alter their fitness. The fluorescence status of the intermediate and final strains was verified by flow cytometry. The flow cytometry outputs clearly displayed (i) the absence of fluorescence signals in the parental strain CEC4591, (ii) a shift toward the mono-GFP gate upon integration of the P*_TDH3_*-*GFP*-*ARG4* cassette in CEC4679, (iii) a shift toward the double-fluorescent BFP/GFP gate upon integration of the P*_TDH3_*-*BFP*-*HIS1* cassette in CEC4685, and (iv) the double-fluorescence status of the population upon integration of the *URA3*-I-*Sce*I-TS cassette in strains CEC5061 and CEC5062 (Fig. S2A).

10.1128/mSphere.00709-18.3 FIG S2 General characterization of strains. (A) Validation of the fluorescence of strains by flow cytometry. A total of 20,000 events per sample were analyzed. (B) Doubling times of C. albicans strains used throughout this study in YPD at 30°C. Download FIG S2, TIF file, 0.5 MB. Copyright © 2019 Marton et al. 2019 Marton et al. This content is distributed under the terms of the Creative Commons Attribution 4.0 International license.

Growth curves performed demonstrated that only the insertion of the *URA3*-I-*Sce*I TS cassette in strains CEC5061 and CEC5062 resulted in a higher growth rate, almost certainly due to the uridine prototrophy in these strains, as *URA3* deletion has been shown to result in significant decreases in C. albicans growth rate even when *ura3Δ* strains are grown in rich medium (21) (Fig. S2B).

### Validation of the I-*Sce*I DNA DSB induction system on Chr7 by 5-FOA counterselection.

As the I-*Sce*I TS is associated with the genetic auxotrophic marker *URA3*, we could assess the frequency of cells that have survived an I-*Sce*I-induced DNA DSB at the TS by monitoring the frequency of appearance of 5-fluoroorotic acid-resistant (5-FOA^R^) colonies upon I-*Sce*I induction. Indeed, 5-FOA^R^ colonies should have lost the *URA3* genetic marker (uridine auxotrophy) and are likely to represent cells that have sustained an I-*Sce*I-dependent DNA DSB through a LOH event, even though point mutations in the *URA3* gene cannot be excluded. We obtained 501 times more 5-FOA^R^ colonies after I-*Sce*I induction when the I-*Sce*I TS was located on Chr7A and 547 times more 5-FOA^R^ colonies when the I-*Sce*I TS was located on Chr7B (Fig. 1B). These data confirmed the efficiency of the I-*Sce*I-dependent DNA DSB induction system on Chr7. The majority of I-*Sce*I DNA DSB-induced 5-FOA^R^ colonies (90 to 100%) resulted from DNA DSB repair by gene conversion, as suggested by fluorescence and auxotrophy profiles of 32 Ura^−^ colonies (Fig. 1C). Similar to what has been observed for Chr4 (13), DNA DSBs by I-*Sce*I on Chr7 are predominantly repaired by gene conversion repair mechanisms, resulting in short-range LOH events.

### An I-SceI-induced DNA DSB on Chr7B leads to viable cells homozygous for the right arm of Chr7A.

Although the 5-FOA assays yield information on the overall occurrence of LOH events encompassing the *URA3* gene, it does not allow us to efficiently study the underrepresented long-range LOH events. Thus, we also investigated LOH frequency upon I-*Sce*I expression using flow cytometry, an assay that specifically detects long-range LOH events. As expected, upon induction of I-*Sce*I in strain CEC5062, possessing the I-*Sce*I TS on the GFP-bearing Chr7B, we observed a 12-fold increase in the appearance of mono-BFP cells (Fig. 1D). We also observed a fivefold increase in the appearance of mono-GFP cells (Fig. 1D). A subset of each population was recovered by fluorescence-activated cell sorting (FACS) and further characterized. We observed that, while the majority of the mono-BFP population included true mono-BFP cells displaying complete Chr7A homozygosis distal to the I-*Sce*I TS, 100% of the rare true mono-GFP cells displayed only partial homozygosis of Chr7B. From a mechanistic point of view, the mono-BFP cells resulted most likely from the repair of the DNA DSBs by mechanisms of break-induced replication or mitotic crossover, while the mono-GFP cells could be one of the possible outcomes of DNA DSB repair by gene conversion with crossover during the G_2_ phase of the cell cycle (Fig. 1E).

### Absence of recovery of cells being fully homozygous for the right arm of Chr7B.

Unlike targeting the right arm of Chr7B, a DNA DSB on the right arm of Chr7A in strain CEC5061 should lead to a higher increase in frequency of the mono-GFP cells compared to the mono-BFP cells (Fig. 1D). Although an augmentation in frequency of both mono-BFP and mono-GFP cells was obtained, the mono-BFP cells still appeared at a higher frequency, 8 × 10^−3^ (± 8 × 10^−4^), compared to 1 × 10^−4^ (± 3 × 10^−5^) for the mono-GFP cells in the induced condition (Fig. 1D). Characterization of a subset of FACS-sorted mono-BFP cells confirmed that the true mono-BFP cells had arisen from a DNA DSB repaired by gene conversion with crossover during G_2_. Further characterization of FACS-sorted mono-GFP cells (corresponding to the expected fluorescence) revealed that the I-*Sce*I-induced homozygosis of Chr7B was only partial in the targeted region. Thus, rather than having experienced break-induced replication or mitotic crossover events extending from the I-*Sce*I site to the BFP/GFP locus, the rare mono-GFP individuals were likely to have resulted from DNA DSB repair by gene conversion with crossover during G_2_ (Fig. 1E). In conclusion, we were unable to recover a single individual that had undergone complete homozygosis of the right arm of Chr7B distal to the I-*Sce*I TS. This suggests that complete homozygosis of the right arm of Chr7B is associated with lethality in the SC5314 genetic background, thus confirming the chromosome homozygosis bias previously observed and validating the hypothesis that the presence of RLA(s) in this region could be a cause.

### A data mining strategy identifies a heterozygous mutation in the *MTR4* gene as a possible cause of the homozygosis bias of Chr7.

**FIG 2:**
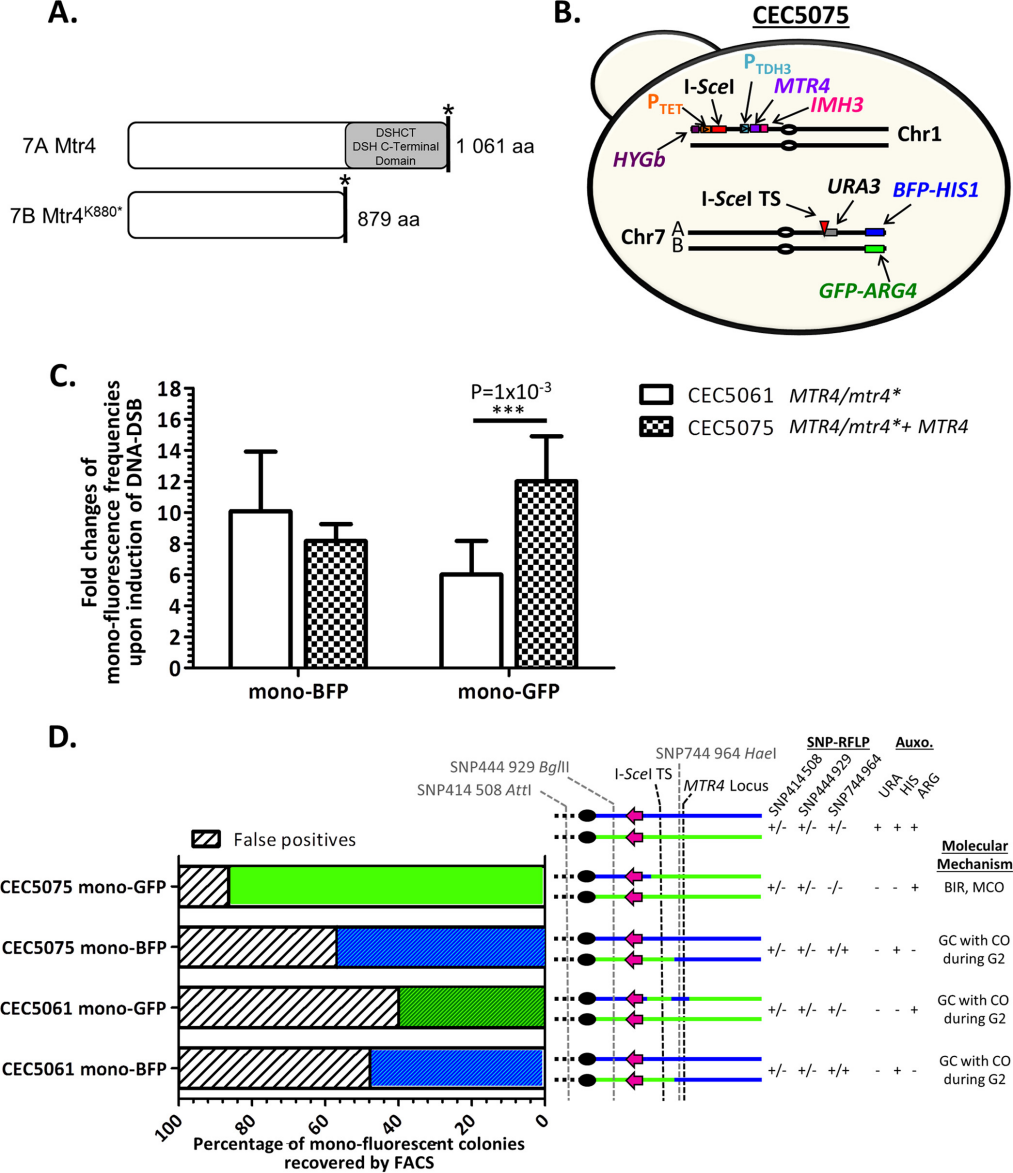
Complementation with a functional *MTR4* allele restores viability of Chr7B homozygous cells. (A) Schematic alignment of the wild-type *MTR4* and mtr4^K880*^-encoded proteins. *MTR4* encodes an ATP-dependent RNA-helicase, and the nonsense mutation in the mtr4^K880*^ allele leads to loss of the DSH C-terminal domain shared by DEAD box helicases. aa, amino acids. (B) Illustration of the CEC5075 complemented strain possessing the functional *MTR4* allele at the *RPS1* locus on Chr1. Centromeres are indicated by ovals. (C) Comparison of the average fold increase of the monofluorescent populations upon DNA DSB induction (ATc/YPD) in both parental (CEC5061) and complemented (CEC5075) strains (*n* = 6) (plus S.D.), significance was determined using a bilateral *t* test (*P* value). (D) Characterization of monofluorescence-sorted individuals (*n* = 32) from induced CEC5061 (I-*Sce*I TS on HapA) and *MTR4-*complemented CEC5075 strains. See the legend to Fig. 1E for explanation.

Genome sequence data obtained from a collection of 182 C. albicans isolates (7), including the reference strain SC5314, was used to compile all heterozygous SNPs within ORFs of Chr7 and search for SNPs (i) generating a premature STOP codon, (ii) showing a heterozygous genotype in SC5314, (iii) never observed in the homozygous state in the collection of 182 genomes, and (iv) located in a coding region never found to be dispensable in C. albicans. Only one such SNP was identified, located at position 746,359 on Chr7B. In C. albicans strain SC5314, this SNP causes a change from AAA (lysine) on Chr7A to TAA (STOP) on Chr7B in the C7_03400C gene. This gene is the orthologue of S. cerevisiae *MTR4* that encodes an essential ATP-dependent RNA helicase involved in RNA processing in S. cerevisiae (22 – 24). In C. albicans SC5314, the HapA allele of *MTR4* encodes a full-length Mtr4 protein, while the HapB allele carrying the STOP-introducing SNP encodes a truncated Mtr4^K880*^ protein that misses a C-terminal DSHCT domain, common to DEAD box helicases (Fig. 2A).

### The mtr4 ^K880*^ allele is responsible for the Chr7 homozygosis bias.

To validate that the identified RLA candidate mtr4^K880*^ is truly responsible for the Chr7 homozygosis bias, the full-length *MTR4* ORF was placed ectopically under the control of the P*_TDH3_* constitutive promoter in strain CEC5061, giving rise to strain CEC5075 (Fig. 2B). Results shown in Fig. 1B and C confirmed that the DNA DSB-inducing system was functional in this strain and that the I-*Sce*I-induced DNA DSB was predominantly repaired by gene conversion as observed in other instances. Strikingly, flow cytometry analysis revealed a significant elevation in the fold increase of mono-GFP cells obtained upon induction of I-*Sce*I in strain CEC5075 by comparison to CEC5061 (Fig. 2C). Upon cell sorting and characterization of these mono-GFP cells, the majority (93%) appeared as true mono-GFP individuals that had become fully homozygous for Chr7B from the I-*Sce*I TS to the BFP/GFP LOH reporter system. These cells were likely to have resulted from the repair of the induced DNA DSB by break-induced replication or mitotic crossover, indicating that complete homozygosis of the right arm of Chr7B is compatible with viability upon addition of a functional *MTR4* allele (Fig. 2D). Similar to what was observed with the CEC5061 parental strain, I-*Sce*I induction in the CEC5075 *MTR4*-complemented strain resulted in an augmentation of mono-BFP cells (Fig. 2C). Eighty-eight percent of these mono-BFP cells corresponded to cells where the DNA DSB had been repaired by gene conversion with crossover during G_2_ (Fig. 2D).

### Upon DNA DSB, major repeat sequences are a source of interhomolog recombination.

**FIG 3:**
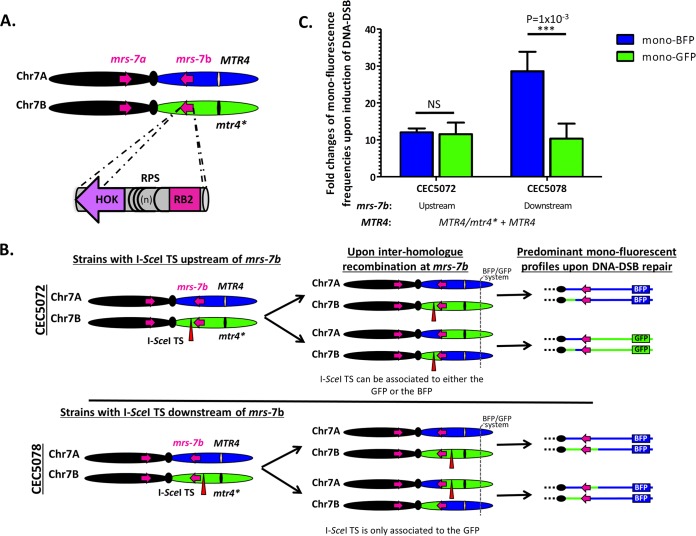
Upon DNA DSB, major repeat sequences are a source of interhomolog recombination. (A) Three subunits compose the MRS, which are hot spots for mitotic crossover. (B) Engineering of strains where the I-*Sce*I TS is located on HapB upstream (CEC5072) or downstream (CEC5078) of *mrs*-*7b*. Illustration of the right arm of Chr7 upon interhomolog recombination at *mrs-7b* followed by the predominant monofluorescence profiles upon DNA DSB repair leading to long-range LOH events. (C) Histogram showing average fold changes (*n* = 6) (plus S.D.) of monofluorescent populations upon I-*Sce*I induction (ATc versus YPD). While no significant difference in augmentation of both populations is detected when the I-*Sce*I TS is placed upstream of *mrs-7b* (CEC5072), a significant difference is observed when the I-*Sce*I TS is placed downstream of *mrs-7b* (CEC5078). Significance was determined using a bilateral *t* test (*P* value).

Our initial strategy for unveiling RLAs was to induce a DNA DSB downstream of *mrs*-*7b* (the MRS located on the right arm of Chr7 [Fig. 3A]) in order to ensure that the repeat sequences would not interfere with the DNA repair mechanisms. To seek validation of this initial assumption, the I-*Sce*I TS was moved upstream of *mrs*-*7b*, between the centromere and *mrs-7b*, on either HapA or HapB and in the presence of an ectopic copy of *MTR4* (Fig. 3B). By plating on 5-FOA-containing medium, we showed that the inducible I-*Sce*I DNA DSB system was functional in the new location and that gene conversion was the preferred mechanism of repair, independently of the targeted haplotype (Fig. 1B and C). Notably, when the I-*Sce*I TS was localized on HapB upstream of *mrs-7b* in an *MTR4-*complemented strain (CEC5072), induction of I-*Sce*I expression resulted in almost equal increases in the mono-GFP and mono-BFP cell populations relative to noninduced conditions (Fig. 3C). This contrasted to what was observed when the I-*Sce*I TS was localized on HapB downstream of *mrs-7b* in an *MTR4-*complemented strain (CEC5078), whereby a greater increase in the mono-BFP cell population than mono-GFP cell population was observed upon I-*Sce*I induction (Fig. 3C). This observation could be explained by interhomolog recombination at *mrs-7b*, which would link the I-*Sce*I TS to either the BFP or GFP markers when the TS is located upstream of *mrs-7b* (Fig. 3B). This would result in equal proportions of mono-BFP and mono-GFP cells upon repair of the I-*Sce*I-induced DNA DSB, keeping in mind that break-induced replication and mitotic crossover are the predominant molecular mechanisms leading to long-range LOH (Fig. 3C). In contrast, when the I-*Sce*I TS is located downstream of *mrs-7b*, it remains linked to the GFP marker, regardless of interhomolog recombination at *mrs-7b*, thus predominantly yielding mono-BFP cells upon I-*Sce*I-induced DNA DSB repair (Fig. 3B and C). Overall, our data suggest that the MRS is a hot spot for interhomolog recombination upon DNA repair on the right arm of Chr7.

Of interest, C. albicans Chr7 is characterized by the occurrence of a second MRS on the left arm, namely, *mrs-7a* (Fig. 3A). Strains possessing the I-*Sce*I TS between the centromere and *mrs-7a* and the BFP/GFP LOH reporter system on the left arm of Chr7 also exhibited an equal rate in mono-BFP and mono-GFP cells upon I-*Sce*I induction, whatever the location of the I-*Sce*I TS on HapA or HapB (data not shown), suggesting an increase in the number of cells that have undergone crossover at *mrs-7a*, linking the I-*Sce*I TS to either the BFP or GFP marker. Thus, *mrs-7a* also appears to be a hot spot for interhomolog recombination on Chr7.

## DISCUSSION

Previous studies have shown that C. albicans strains harbor recessive lethal alleles (RLAs) that are responsible for a bias upon homozygosis of certain chromosomes, whereby only one of the two homologs can be retained in the homozygous state (Table 1). Yet, the nature of these RLAs is generally unknown. In this report, we have ascribed the Chr7 homozygosis bias of the C. albicans laboratory strain SC5314 to a heterozygous SNP introducing a premature STOP codon in the *MTR4* gene. Furthermore, we have unveiled the contribution of the major repeat sequence (MRS) to interhomolog recombination and hence, chromosome dynamics in C. albicans.

**TABLE 1:** Summary of chromosome homozygosis bias observed in the literature

Chr	Parasexuality^*a*^	*RAD52* mutants^*b*^	Obligate diploid^*c*^	Bias summary	
R	No BB	No BB		None	
1	No AA, no BB	No BB	No BB		BB bias
2	No BB			None	
3	No AA	No AA	No BB	None	
4	No AA, no BB	No BB	No BB		BB bias
5		No AA		None	
6	No BB	No BB	No BB		BB bias
7	No BB	No BB	No BB		BB bias

a Data from reference 17.

b Data from reference 18.

c Data from reference 8.

Our work focused on the homozygous bias observed for Chr7, suggesting the presence of at least one RLA on Chr7B (Table 1). Using a fluorescence-based LOH reporter system and an I-*Sce*I-dependent DNA DSB-inducing system, we have shown that while long-range homozygosis of Chr7A does not affect cell viability, long-range homozygosis of Chr7B is nonviable in C. albicans strain SC5314. A library of SNPs compiled from 182 clinical C. albicans genomes (7) was searched for SNPs that would result in the most drastic outcome, i.e., a premature STOP codon. In this respect, we surely underestimated the presence of recessive lethal alleles, as mutations other than premature STOP-introducing SNPs are likely to result in lethal phenotypes. Indeed, nonsynonymous SNPs in coding regions have been shown to negatively impact protein function or regulation (25, 26). Our approach pinpointed an SNP in the C. albicans *MTR4* gene that complementation experiments confirmed to be the RLA responsible for the lethality of individuals homozygous for Chr7B in strain SC5314. The identified SNP results in a truncated form of Mtr4 that lacks the last 182 C-terminal amino acids, encompassing a DEAD box family DSHCT domain (Mtr4^K880*^ [Fig. 2A]). In S. cerevisiae, *MTR4* encodes an ATP-dependent RNA helicase whose deletion results in nuclear accumulation of unprocessed RNAs (27), and reduction of function results in increased sensitivity to both benomyl and nocodazole (28). Importantly, it has been shown that the C-terminal domain of other fungal RNA helicases is critical for their proper RNA-unwinding function (29), consistent with mtr4^K880*^ being a loss-of-function allele and homozygosity of this allele being lethal.

**FIG 4:**
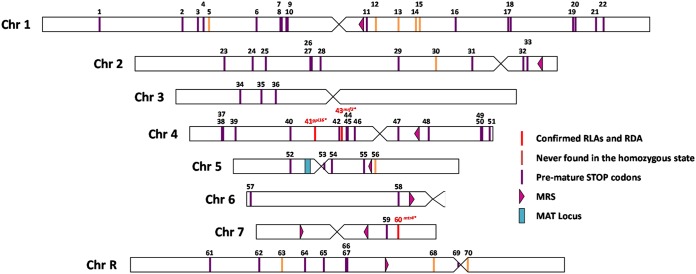
Schematic representation of the localization of premature STOP-inducing SNPs in the C. albicans SC5314 genome. Representation of 70 heterozygous SNPs (vertical bars) inducing a premature STOP codon distributed on all eight chromosomes of the reference strain SC5314. SNPs inducing premature STOP codons that are never found in the homozygous state in the library of 182 genomes of clinical C. albicans isolates are represented in orange, while confirmed recessive lethal and deleterious alleles are identified in red. Details regarding each SNP can be found in Table S1 using the assigned numbers in this figure.

Our study and that of Feri et al. (13) indicate that the mining of the genomes of a large panel of C. albicans isolates for premature STOP-introducing SNPs is a suitable approach to identify RLAs responsible for chromosome homozygosis bias in C. albicans strains. As a reference, we provide a list of 70 genes that harbor a STOP-introducing SNP in one of the two alleles in the laboratory strain SC5314 in Table S1 in the supplemental material, including 12 alleles (Table 2) that were never found in the homozygous state in a collection of 182 genome-sequenced isolates representative of the C. albicans population (7). The locations of these 12 candidate RLAs across the C. albicans genome are shown in Fig. 4. Subsequent Sanger sequencing will be necessary to confirm these alleles. Our reduced number of genes with SNPs introducing a premature STOP codon in strain SC5314 compared to Muzzey et al. (10) could be explained by the stringency of our analysis. Indeed, only positions with high SNP quality and coverage depth of >20 for all 182 strains of our collection were considered for further analysis.

**TABLE 2:**
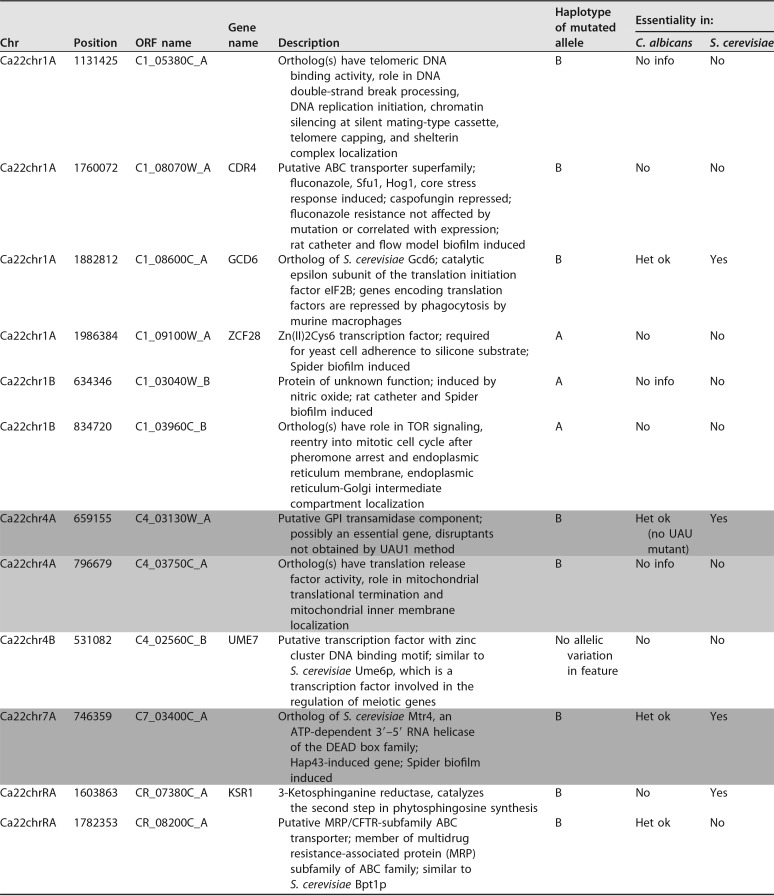
Genome-wide RLA candidates in the C. albicans strain SC5314^*a*^

a Confirmed RLAs are indicated by medium gray shading (reference 13 and this study), and the recessive deleterious allele is indicated by light gray shading (13).

10.1128/mSphere.00709-18.4 TABLE S1 List of heterozygous SNPs inducing premature STOP codons in the C. albicans SC5314 genome. Download Table S1, DOCX file, 0.04 MB. Copyright © 2019 Marton et al. 2019 Marton et al. This content is distributed under the terms of the Creative Commons Attribution 4.0 International license.

A promising candidate to explain the homozygosis bias observed for Chr1 is the gcd6* allele (C1_08600C_B) that carries a SNP introducing a premature STOP codon, truncating the protein from the 17 C-terminal amino acids. In S. cerevisiae, *GCD6* encodes the catalytic epsilon subunit of the translation initiation factor eIF2B. The truncated domain is found at the C terminus of several translation initiation factors and is important for mediating protein-protein interactions. *GCD6* is essential in S. cerevisiae, as null mutants are not viable. The same is likely to be true in C. albicans, since heterozygous mutants are viable, but null mutants have not been reported in the literature. Therefore, it can be hypothesized that homozygosis of gcd6*, located on Chr1B, could result in cell death and be responsible for the homozygosis bias observed on Chr1 (Table 1).

Interestingly, a recent transposon mutagenesis screen in haploid C. albicans also argues that Ca*GCD6* is essential (30). Among the 12 candidate RLAs presented in Table 2, the same screen argues for essentiality of Ca*GPI16* (previously identified by Feri et al. [13]) and Ca*MTR4* (identified in this study). Although the 10 remaining genes in Table 2 were defined as nonessential genes by Segal et al. (30), it is puzzling to see that the mutated alleles with a premature STOP codon were never observed in the homozygous state in the natural population of 182 C. albicans isolates. This observation highlights the complementarity of the study by Segal et al. (30) and our work, given that gene essentiality can vary depending on growth conditions and *in vitro* assessment of gene essentiality does not necessarily correlate with *in vivo* essentiality.

From a mechanistic point of view, our work revealed that most of the cells that have undergone an I-*Sce*I-mediated DNA DSB on Chr7 use gene conversion as a repair mechanism, thus limiting LOH extent and the loss of genetic information. Break-induced replication, mitotic crossover, gene conversion with crossover and chromosome truncation that lead to long-range LOH events in decreasing order are less frequently utilized. Our results are consistent with those of Feri et al. (13) on Chr4, arguing that our observation is not locus specific but can be applied genome-wide in C. albicans. However, we cannot exclude the possibility that the relative usage of these repair mechanisms is specific to I-*Sce*I-induced DNA DSB, i.e., DNA DSBs induced by the CRISPR-*Cas9* RNA-guided endonuclease could be preferentially handled by another mechanism of DNA repair.

In the course of this study, we also addressed the role that repetitive sequences, such as MRS, might play on the overall genome dynamics of C. albicans. This was achieved by studying how MRS position affects the outcome of I-*Sce*I-induced LOH. Even though most C. albicans chromosomes possess a unique MRS region, the presence of two MRS regions, one on each arm, is unique to Chr7 (19). We observed that the presence of *mrs-7b* or *mrs-7a* between the I-*Sce*I TS and the telomere-proximal LOH reporter system on Chr7 results in equal augmentation of the mono-BFP and mono-GFP populations upon I-*Sce*I-dependent DNA DSB (Fig. 3). This contrasts with what is observed when the I-*Sce*I TS is located downstream of the MRS, whereby the monofluorescent population arising by break-induced replication or mitotic crossover is increased relative to the monofluorescent population arising by gene conversion with crossover in the G_2_ phase of the cell cycle (a scarce molecular mechanism), unless a RLA is present. This suggests that the MRS could be a hot spot for interhomolog mitotic crossover on Chr7. Indeed, upon recombination events at the MRS, DSBs repaired by break-induced replication or mitotic crossover would result in a relatively equal appearance of both monofluorescent populations when I-*Sce*I-TS is located between the centromere and the MRS (Fig. 3B and C).

Mitotic crossovers at MRS could be an intrinsic feature of these repeat regions or be triggered by either (i) stress resulting from I-*Sce*I overexpression or (ii) the physical I-*Sce*I-induced DNA DSB. The former would imply that increased mitotic crossovers at MRS should be expected on all chromosomes remaining to be tested. The latter would imply that increased mitotic crossovers at Chr7 MRS are observed only upon repair of a DNA DSB upstream of these repeated regions. These latter hypotheses are consistent with a role for stress in the enhancement of the recombination frequency near the MRS or in general, as already suggested by Lephart et al. (20). Importantly, our results confirm the original proposal of Pujol et al. (31) that MRS on Chr7 are hot spots for recombination. Concretely, MRS would allow switching C. albicans haplotypes by generating a new combination of alleles. Information regarding the biological importance of MRS remains scarce despite the positive selection on MRS leading to the maintenance of these large and unique repeats in the C. albicans genome. Such recombination events provide C. albicans with increased opportunities to survive DNA DSBs whose repair can lead to homozygosis of recessive lethal or deleterious alleles. This might explain the maintenance of MRS in this species.

## MATERIALS AND METHODS

### Strains and media.

C. albicans strains used in this work are derived from the reference strain SC5314. The cloning experiments were carried out using One Shot TOP10 chemically competent Escherichia coli cells (ThermoFisher Scientific). All C. albicans strains and E. coli plasmids generated and used throughout this investigation are listed in Table 3 and Table S2 in the supplemental material, respectively.

**TABLE 3:**
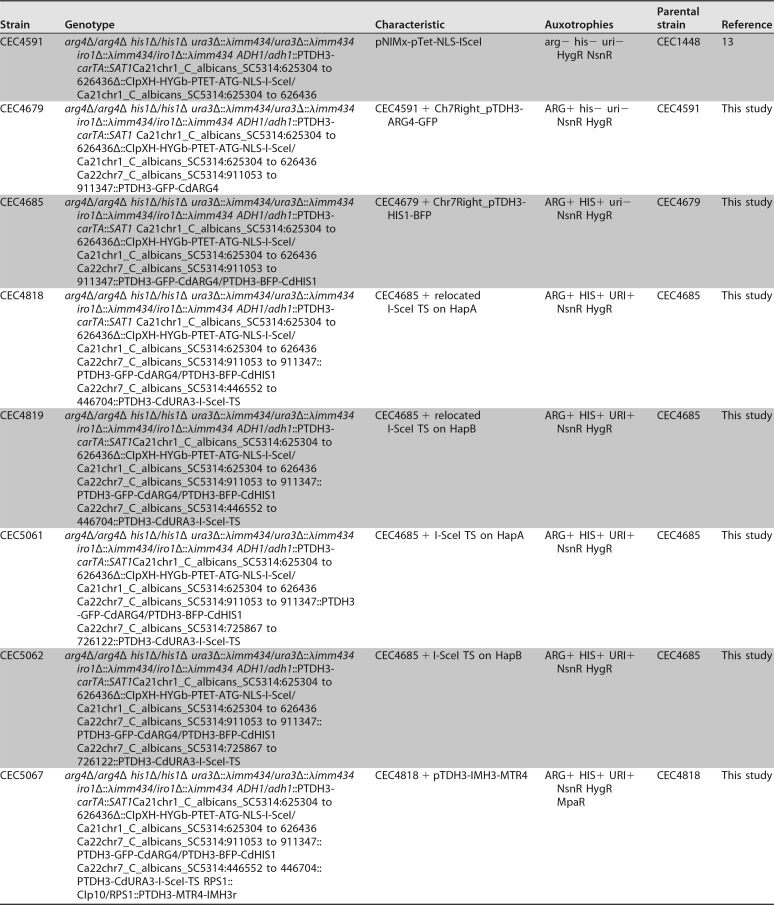

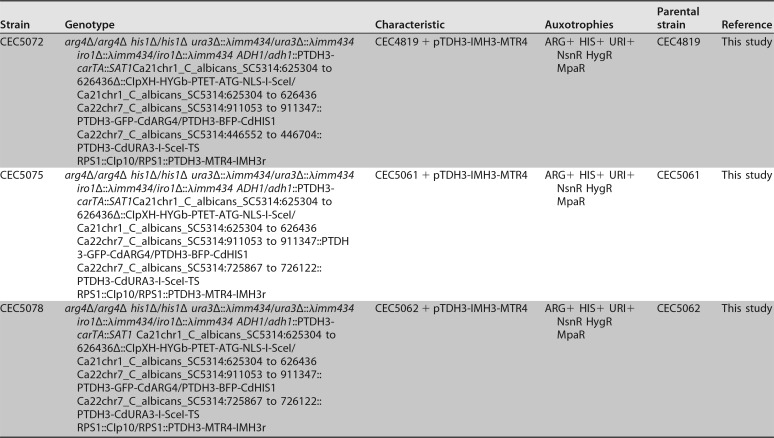
C. albicans strains used in this study

10.1128/mSphere.00709-18.5 TABLE S2 Plasmids used in this study. Download Table S2, DOCX file, 0.03 MB. Copyright © 2019 Marton et al. 2019 Marton et al. This content is distributed under the terms of the Creative Commons Attribution 4.0 International license.

C. albicans cells were maintained on rich yeast extract-peptone-dextrose (YPD) medium (1% yeast extract, 2% peptone, 2% dextrose). Synthetic complete (SC) (0.67% yeast nitrogen base without amino acids, 2% dextrose, 0.08% drop out mix) and synthetic defined (SD) (0.67% yeast nitrogen base without amino acids, 2% dextrose) media, both with drop out amino acids, depending on auxotrophy of the strains, were used as selective media. Solid media were obtained by adding 2% agar. 5-Fluoroorotic acid (5-FOA)-containing agar medium (0.67% yeast nitrogen base without amino acid, 0.0625% 5-fluoroorotic acid [Toronto Research Chemicals], 0.01% uridine, 2% glucose, 2% agar supplemented with arginine and histidine) was used to detect cells that have an nonfunctional *URA3* gene. Mycophenolic acid (MPA) medium is composed of SD medium with 5 μg/ml MPA (Sigma-Aldrich). Meanwhile, E. coli strains were cultured on/in LB or 2YT medium, with appropriate antibiotics for selection purposes (kanamycin [50 μg/ml], ticarcillin [50 μg/ml], and gentamicin [10 μg/ml]).

### *In silico* identification of potential recessive lethal alleles.

A collection of 182 C. albicans genomes (7) was utilized with the intention to search for potential RLAs. SNPs were compiled and filtered with the following criteria: (i) an SNP that introduces a premature STOP codon in a coding region (open reading frame [ORF]), (ii) this SNP was never observed in the homozygous state, (iii) the SNP lies in a coding region never found to be dispensable in C. albicans, and (iv) the SNP was found in the heterozygous state in the reference strain SC5314.

### Plasmid constructions.

Methods for the construction of plasmids used for I-*Sce*I target sequence (TS) insertion (pFA-*URA3*-I*Sce*I-TS-*HSP90*/*CUP9*) and for complementation with the wild-type *MTR4* allele (CIp10-P*_TDH3_*-*MTR4*-*IMH*3) are described in Text S1 in the supplemental material.

10.1128/mSphere.00709-18.1 TEXT S1 Supplemental methods. Plasmid constructions and *Candida albicans* strain constructions. Download Text S1, DOCX file, 0.05 MB. Copyright © 2019 Marton et al. 2019 Marton et al. This content is distributed under the terms of the Creative Commons Attribution 4.0 International license.

### C. albicans strain constructions.

Construction of strain CEC4685 is described in Text S1. Briefly, this strain is derived from the common laboratory strain SN76 and carries the I-*Sce*I-encoding gene under the control of the inducible P*_TET_* promoter (32), pNIMX coding for the transactivator necessary for activation of the P*_TET_* promoter in the presence of tetracycline derivatives (33), and the BFP/GFP LOH reporter system (15) on the right arm of Chr7. Integration of the I-*Sce*I TS was achieved by transformation of strain CEC4685 with a PCR-amplified cassette bearing the I-*Sce*I TS and Chr7 homology regions, the latter borne from primers 9 and 10 (Table S3) used in amplification of pFA-*URA3*-I*Sce*I-TS-*CDR3*/TG(GCC)2 (13). This led to prototroph strains named CEC5061 (I-*Sce*I TS on HapA) and CEC5062 (I-*Sce*I TS on HapB) (Table 3).

10.1128/mSphere.00709-18.6 TABLE S3 Primers used in this study. Download Table S3, DOCX file, 0.03 MB. Copyright © 2019 Marton et al. 2019 Marton et al. This content is distributed under the terms of the Creative Commons Attribution 4.0 International license.

Relocalization of I-*Sce*I TS upstream of the MRS was conducted by transforming strain CEC4685 with *Hpa*I+*Sca*II-cut plasmid of pFA-*URA3*-I*Sce*I-TS-*HSP90/CUP9*, which carries the *URA3*-I-*Sce*I TS construction for integration on Chr7. The now prototroph transformants were selected on SD medium. The final strains were named CEC4818 (I-*Sce*I TS on HapA) and CEC4819 (I-*Sce*I TS on HapB) (Table 3).

Strains possessing the BFP/GFP LOH reporter system on the left arm of Chr7 were generated by positioning the heterozygous BFP/GFP locus close to the left telomere (position 108,458 to 108,838) and the I-*Sce*I TS at position 367,060 to 367,145 between *mrs-7a* (position 228,342 to 242,083) and the centromere (position 425,808 to 428,708).

Complementation of strains CEC5061, CEC5062, CEC4818, and CEC4819 with the wild-type *MTR4* allele at the *RPS1* locus was achieved using the *Stu*I-linearized CIp10-P*_TDH3_*-*MTR4*-*IMH3* plasmid described in Text S1. Transformants were selected on SD medium containing 5 µg/ml MPA (Sigma-Aldrich), and the final strains were named CEC5067, CEC5072, CEC5075, and CEC5078, respectively (Table 3).

Following each transformation step, junction PCRs were conducted to ensure proper integrations. Additional controls were conducted such as fluorescence check (flow cytometry of 20,000 cells) and auxotrophy testing of strains (by spotting on SD supplemented with either Arg, His, Ura, or MPA). To ensure that the strains generated in the course of this study did not display growth defects resulting from transformation events, growth curves were generated using a TECAN Sunrise with OD readings (620 nm) every 10 min for a period of 48 h. Doubling times were outputted from the generated OD values and analyzed using GraphPad Prism 5.

### Induction of the *Tet*-On system.

In order to activate the *Tet*-On promoter and achieve I-*Sce*I protein production, single colonies were precultured in liquid SC-His-Arg medium at 30°C. After overnight growth, induction was conducted in YPD plus anhydrotetracycline (ATc) (3 µg/ml) (Thermofisher ACROS Organics) for 8 h at 30°C, followed by an overnight recovery in YPD.

### 5-Fluoroorotic acid selection.

Following the I-*Sce*I induction protocol, as seen above, three different cell dilutions of cultures (20,000 cells, 2,000 cells, and 200 cells) grown in the presence (induced) or absence (noninduced; control) of ATc were plated on 5-fluoroorotic acid (5-FOA)-containing plates in triplicates. Dilutions were verified by plating a volume corresponding to 100 cells on YPD plates. The plates were incubated at 30°C for 3 days before analysis.

### Cell preparation for flow cytometry and analysis.

All flow cytometry analyses were conducted on the MACSQuant analyzer (Miltenyi Biotec) where BFP is detected with a 405-nm laser and 425- to 475-nm filters and GFP is detected with a 488-nm laser and 500- to 550-nm filters. Data for a maximum of 10^6^ cells were analyzed using the FlowJo V10.1 software. The gates to determine the LOH frequencies were arbitrarily selected but conserved throughout sample analysis.

### Cell sorting.

Induced and noninduced cultures were filtered using BD Falcon Cell strainers in order to remove large debris and filamentous cells that could obstruct the tubing system of the cytometer. The MoFlo Astrios flow cytometer was used to analyze and sort the cells of interest. For each sorted gate, 1,000 cells were recovered in 400 μl of liquid YPD medium, plated immediately after cell sorting on four YPD petri plates, and incubated at 30°C for 48 h before collection of results.

### SNP-RFLP.

*In silico* identification of heterozygous SNPs affecting a restriction site on only one haplotype of Chr7 was used for haplotype characterization of strains. First, a nucleotide multiple sequence alignment by MUSCLE was conducted using the Chr7A and Chr7B sequences from the reference strain SC5314 (22). Heterozygous SNPs were selected using the following criteria. (i) It interrupts a commonly known restriction site on one haplotype. (ii) The selected enzyme does not cut again within a range of 1 kb. (iii) The heterozygous SNP is present in most strains of the collection of 182 C. albicans clinical strains (7). Second, primer pairs were designed to result in PCR products with different digestion profiles and used to verify the presence of the heterozygous SNP in our strain of interest.

These SNPs were used for two distinct purposes: (i) to assign the Chr7 homolog targeted by I-*Sce*I TS integration and (ii) to monitor the heterozygous status of the left and right arms of Chr7 upon DNA DSB repair. Regions of roughly 2.5 kb surrounding the heterozygous SNPs were amplified by PCR and digested with the appropriate restriction enzyme overnight. C. albicans gDNA extractions, PCRs, and amplicon verifications were conducted following the methods of Feri et al. (13), while a list of the primers used can be found in Table S3. The SNPs at positions 444,929 (*Bgl*II cutting HapA) and 727,328 (*Hpa*I cutting HapA) were used to identify the I-*Sce*I-targeted haplotype of the right arm of Chr7, when the I-*Sce*I TS is located upstream and downstream of the *mrs-7b*, respectively. The 2.2-kb and 2.16-kb regions around the heterozygous SNPs located at positions 414,508 (left arm) and 744,964 (right arm) utilizing *Att*I (cutting HapB) and *Hae*II (cutting HapB) enzymes, respectively, were used to assess the heterozygous status of both Chr7 arms. All SNP-RFLP sites and the enzyme-sensitive haplotypes are summarized in Fig. S1.

## References

[1] Gerstein AC, Kuzmin A, Otto SP 2014 Loss-of-heterozygosity facilitates passage through Haldane’s sieve for *Saccharomyces cerevisiae* undergoing adaptation. Nat Commun 5:3819. doi:10.1038/ncomms4819. 24804896

[2] Coste A, Selmecki A, Forche A, Diogo D, Bougnoux M-E, d'Enfert C, Berman J, Sanglard D 2007 Genotypic evolution of azole resistance mechanisms in sequential *Candida albicans* isolates. Eukaryot Cell 6:1889–1904. doi:10.1128/EC.00151-07. 17693596 PMC2043391

[3] Selmecki A, Forche A, Berman J 2006 Aneuploidy and isochromosome formation in drug-resistant *Candida albicans*. Science 313:367–370. doi:10.1126/science.1128242. 16857942 PMC1717021

[4] Prieto D, Correia I, Pla J, Román E 2016 Adaptation of *Candida albicans* to commensalism in the gut. Future Microbiol 11:567–583. doi:10.2217/fmb.16.1. 27070839

[5] Jones T, Federspiel NA, Chibana H, Dungan J, Kalman S, Magee BB, Newport G, Thorstenson YR, Agabian N, Magee PT, Davis RW, Scherer S 2004 The diploid genome sequence of *Candida albicans*. Proc Natl Acad Sci U S A 101:7329–7334. doi:10.1073/pnas.0401648101. 15123810 PMC409918

[6] Hirakawa MP, Martinez DA, Sakthikumar S, Anderson MZ, Berlin A, Gujja S, Zeng Q, Zisson E, Wang JM, Greenberg JM, Berman J, Bennett RJ, Cuomo CA 2015 Genetic and phenotypic intra-species variation in *Candida albicans*. Genome Res 25:413–425. doi:10.1101/gr.174623.114. 25504520 PMC4352881

[7] Ropars J, Maufrais C, Diogo D, Marcet-Houben M, Perin A, Sertour N, Mosca K, Permal E, Laval G, Bouchier C, Ma L, Schwartz K, Voelz K, May RC, Poulain J, Battail C, Wincker P, Borman AM, Chowdhary A, Fan S, Kim SH, Le Pape P, Romeo O, Shin JH, Gabaldon T, Sherlock G, Bougnoux M-E, d’Enfert C 2018 Gene flow contributes to diversification of the major fungal pathogen Candida albicans. Nat Commun 9:2253. doi:10.1038/s41467-018-04787-4. 29884848 PMC5993739

[8] Hickman MA, Zeng G, Forche A, Hirakawa MP, Abbey D, Harrison BD, Wang Y-M, Su C, Bennett RJ, Wang Y, Berman J 2013 The ‘obligate diploid’ *Candida albicans* forms mating-competent haploids. Nature 494:55–59. doi:10.1038/nature11865. 23364695 PMC3583542

[9] Abbey DA, Funt J, Lurie-Weinberger MN, Thompson DA, Regev A, Myers CL, Berman J 2014 YMAP: a pipeline for visualization of copy number variation and loss of heterozygosity in eukaryotic pathogens. Genome Med 6:1–16. doi:10.1186/s13073-014-0100-8. 25505934 PMC4263066

[10] Muzzey D, Schwartz K, Weissman JS, Sherlock G 2013 Assembly of a phased diploid *Candida albicans* genome facilitates allele-specific measurements and provides a simple model for repeat and indel structure. Genome Biol 14:R97. doi:10.1186/gb-2013-14-9-r97. 24025428 PMC4054093

[11] Gómez-Raja J, Andaluz E, Magee B, Calderone R, Larriba G 2008 A single SNP, G929T (Gly310Val), determines the presence of a functional and a non-functional allele of *HIS4* in *Candida albicans* SC5314: detection of the non-functional allele in laboratory strains. Fungal Genet Biol 45:527–541. doi:10.1016/j.fgb.2007.08.008. 17964203 PMC2605509

[12] Ciudad T, Hickman M, Bellido A, Berman J, Larriba G 2016 Phenotypic consequences of a spontaneous loss of heterozygosity in a common laboratory strain of *Candida albicans*. Genetics 203:1161–1176. doi:10.1534/genetics.116.189274. 27206717 PMC4937476

[13] Feri A, Loll-Krippleber R, Commere P-H, Maufrais C, Sertour N, Schwartz K, Sherlock G, Bougnoux M-E, D’Enfert C, Legrand M 2016 Analysis of repair mechanisms following an induced double strand break uncovers recessive deleterious alleles in the *Candida albicans* diploid genome. mBio 7:e01109-16. doi:10.1128/mBio.01109-16. 27729506 PMC5061868

[14] Muzzey D, Sherlock G, Weissman JS 2014 Extensive and coordinated control of allele-specific expression by both transcription and translation in *Candida albicans*. Genome Res 24:963–973. doi:10.1101/gr.166322.113. 24732588 PMC4032860

[15] Loll-Krippleber R, Feri A, Nguyen M, Maufrais C, Yansouni J, d'Enfert C, Legrand M 2015 A FACS-optimized screen identifies regulators of genome stability in *Candida albicans*. Eukaryot Cell 14:311–322. doi:10.1128/EC.00286-14. 25595446 PMC4346560

[16] Malkova A, Klein F, Leung W-Y, Haber JE 2000 HO endonuclease-induced recombination in yeast meiosis resembles Spo11-induced events. Proc Natl Acad Sci U S A 97:14500–14505. doi:10.1073/pnas.97.26.14500. 11121053 PMC18948

[17] Forche A, Alby K, Schaefer D, Johnson AD, Berman J, Bennett RJ 2008 The parasexual cycle in *Candida albicans* provides an alternative pathway to meiosis for the formation of recombinant strains. PLoS Biol 6:e110. doi:10.1371/journal.pbio.0060110. 18462019 PMC2365976

[18] Andaluz E, Bellido A, Gómez-Raja J, Selmecki A, Bouchonville K, Calderone R, Berman J, Larriba G 2011 Rad52 function prevents chromosome loss and truncation in *Candida albicans*. Mol Microbiol 79:1462–1482. doi:10.1111/j.1365-2958.2011.07532.x. 21272099 PMC3564047

[19] Chibana H, Magee PT 2009 The enigma of the major repeat sequence of *Candida albicans*. Future Microbiol 4:171–179. doi:10.2217/17460913.4.2.171. 19257844

[20] Lephart PR, Chibana H, Magee PT 2005 Effect of the major repeat sequence on chromosome loss in *Candida albicans*. Eukaryot Cell 4:733–741. doi:10.1128/EC.4.4.733-741.2005. 15821133 PMC1087809

[21] Kirsch DR, Whitney RR 1991 Pathogenicity of *Candida albicans* auxotrophic mutants in experimental infections. Infect Immun 59:3297–3300. 1879944 10.1128/iai.59.9.3297-3300.1991 PMC258168

[22] Skrzypek MS, Binkley J, Binkley G, Miyasato SR, Simison M, Sherlock G 2017 The Candida Genome Database (CGD): incorporation of Assembly 22, systematic identifiers and visualization of high throughput sequencing data. Nucleic Acids Res 45:D592–D596. doi:10.1093/nar/gkw924. 27738138 PMC5210628

[23] Giaever G, Chu AM, Ni L, Connelly C, Riles L, Véronneau S, Dow S, Lucau-Danila A, Anderson K, André B, Arkin AP, Astromoff A, El Bakkoury M, Bangham R, Benito R, Brachat S, Campanaro S, Curtiss M, Davis K, Deutschbauer A, Entian K-D, Flaherty P, Foury F, Garfinkel DJ, Gerstein M, Gotte D, Güldener U, Hegemann JH, Hempel S, Herman Z, Jaramillo DF, Kelly DE, Kelly SL, Kötter P, LaBonte D, Lamb DC, Lan N, Liang H, Liao H, Liu L, Luo C, Lussier M, Mao R, Menard P, Ooi SL, Revuelta JL, Roberts CJ, Rose M, Ross-Macdonald P, Scherens B, Schimmack G, et al 2002 Functional profiling of the *Saccharomyces cerevisiae* genome. Nature 418:387. doi:10.1038/nature00935. 12140549

[24] Bernstein KA, Granneman S, Lee AV, Manickam S, Baserga SJ 2006 Comprehensive mutational analysis of yeast DEXD/H box RNA helicases involved in large ribosomal subunit biogenesis. Mol Cell Biol 26:1195–1208. doi:10.1128/MCB.26.4.1195-1208.2006. 16449635 PMC1367183

[25] De Gobbi M, Viprakasit V, Hughes JR, Fisher C, Buckle VJ, Ayyub H, Gibbons RJ, Vernimmen D, Yoshinaga Y, de Jong P, Cheng J-F, Rubin EM, Wood WG, Bowden D, Higgs DR 2006 A regulatory SNP causes a human genetic disease by creating a new transcriptional promoter. Science 312:1215–1217. doi:10.1126/science.1126431. 16728641

[26] Nackley AG, Shabalina SA, Tchivileva IE, Satterfield K, Korchynskyi O, Makarov SS, Maixner W, Diatchenko L 2006 Human catechol-O-methyltransferase haplotypes modulate protein expression by altering mRNA secondary structure. Science 314:1930–1933. doi:10.1126/science.1131262. 17185601

[27] Liang S, Hitomi M, Hu YH, Liu Y, Tartakoff AM 1996 A DEAD-box-family protein is required for nucleocytoplasmic transport of yeast mRNA. Mol Cell Biol 16:5139–5146. doi:10.1128/MCB.16.9.5139. 8756671 PMC231514

[28] Smith SB, Kiss DL, Turk E, Tartakoff AM, Andrulis ED 2011 Pronounced and extensive microtubule defects in a *Saccharomyces cerevisiae* DIS3 mutant. Yeast 28:755–769. doi:10.1002/yea.1899. 21919057 PMC3367412

[29] Mohr G, Del Campo M, Mohr S, Yang Q, Jia H, Jankowsky E, Lambowitz AM 2008 Function of the C-terminal domain of the DEAD-box protein Mss116p analyzed *in vivo* and *in vitro*. J Mol Biol 375:1344–1364. doi:10.1016/j.jmb.2007.11.041. 18096186 PMC2242632

[30] Segal ES, Gritsenko V, Levitan A, Yadav B, Dror N, Steenwyk JL, Silberberg Y, Mielich K, Rokas A, Gow NAR, Kunze R, Sharan R, Berman J 2018 Gene essentiality analyzed by *in vivo* transposon mutagenesis and machine learning in a stable haploid isolate of *Candida albicans*. mBio 9:e02048-18. doi:10.1128/mBio.02048-18. 30377286 PMC6212825

[31] Pujol C, Joly S, Nolan B, Srikantha T, Soll DR 1999 Microevolutionary changes in *Candida albicans* identified by the complex Ca3 fingerprinting probe involve insertions and deletions of the full-length repetitive sequence RPS at specific genomic sites. Microbiology 145:2635–2646. doi:10.1099/00221287-145-10-2635. 10537185

[32] Park Y-N, Morschhäuser J 2005 Tetracycline-inducible gene expression and gene deletion in *Candida albicans*. Eukaryot Cell 4:1328–1342. doi:10.1128/EC.4.8.1328-1342.2005. 16087738 PMC1214539

[33] Chauvel M, Nesseir A, Cabral V, Znaidi S, Goyard S, Bachellier-Bassi S, Firon A, Legrand M, Diogo D, Naulleau C, Rossignol T, d’Enfert C 2012 A versatile overexpression strategy in the pathogenic yeast *Candida albicans*: identification of regulators of morphogenesis and fitness. PLoS One 7:e45912. doi:10.1371/journal.pone.0045912. 23049891 PMC3457969

