# Supplementary figures and images for: Identification of Recessive Lethal Alleles in the Diploid Genome of a Candida albicans Laboratory Strain Unveils a Potential Role of Repetitive Sequences in Buffering Their Deleterious Impact
Source: mSphere. 2019 Feb 13;4(1):e00709-18. doi: 10.1128/mSphere.00709-18 (PMC6374597; doi:10.1128/mSphere.00709-18)

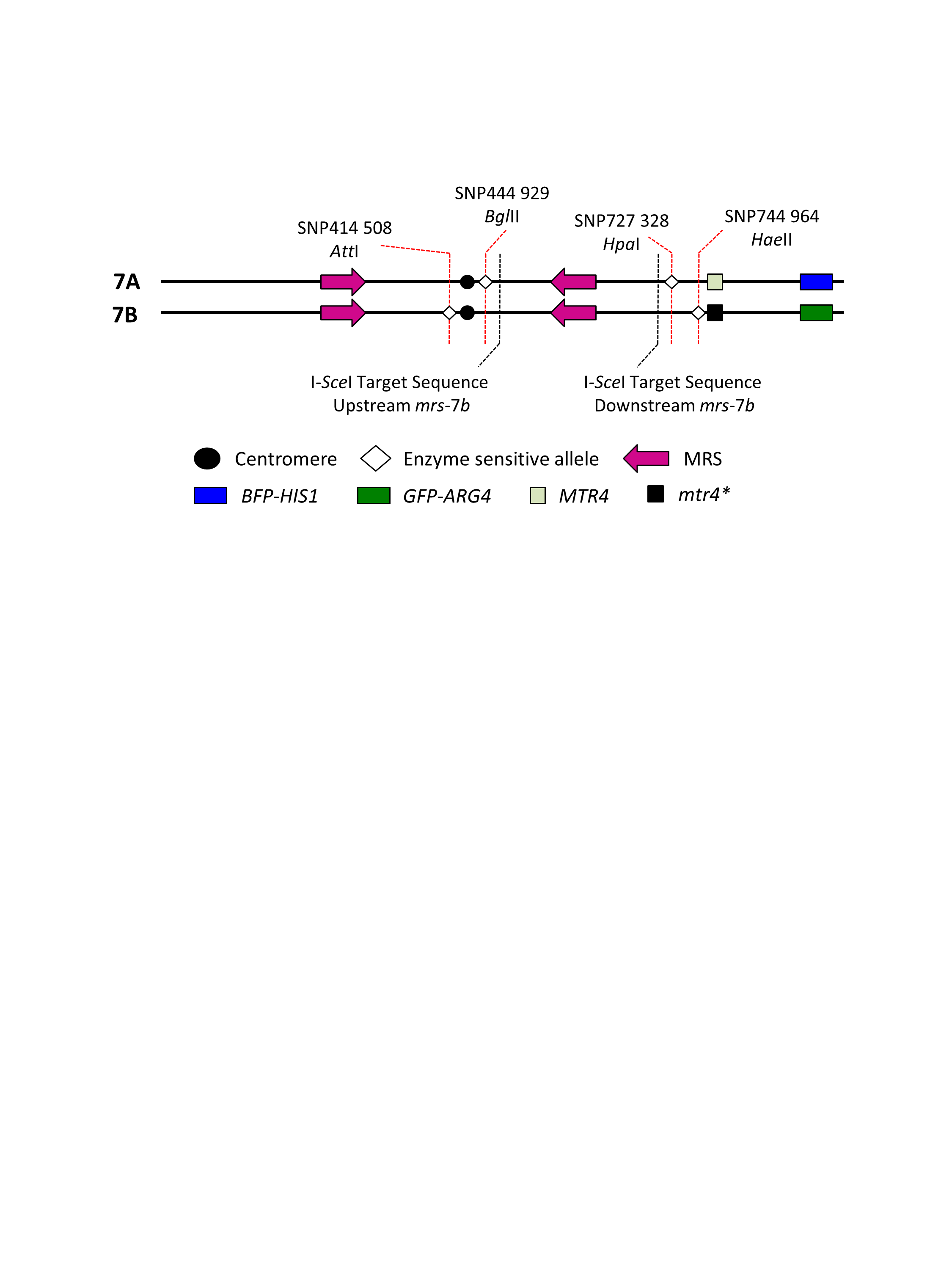

Supplement: FIG S1 [file mSphere.00709-18-sf001.tif]

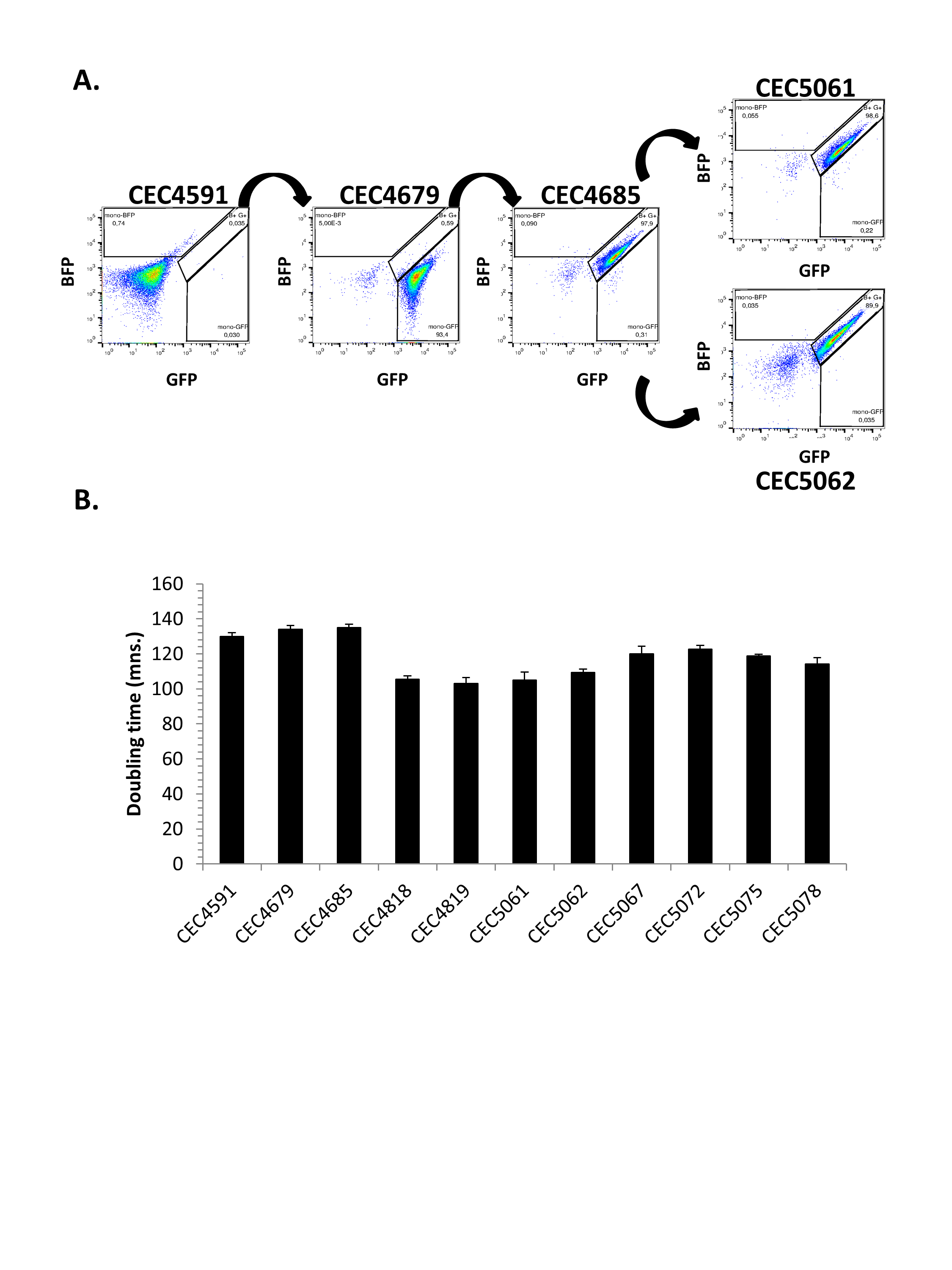

Supplement: FIG S2 [file mSphere.00709-18-sf002.tif]
