# Supplementary material for: Identification of Recessive Lethal Alleles in the Diploid Genome of a Candida albicans Laboratory Strain Unveils a Potential Role of Repetitive Sequences in Buffering Their Deleterious Impact
Source: mSphere. 2019 Feb 13;4(1):e00709-18. doi: 10.1128/mSphere.00709-18 (PMC6374597; doi:10.1128/mSphere.00709-18)
Supplement: TABLE S1 [file mSphere.00709-18-st001.docx]

**Table S1:** **List of heterozygous SNPs inducing premature STOP codons in the *C. albicans* SC5314 genome.**

| **# in Fig. 4** | **Chr-Position** | **Position** | **ORF Name** | | **Gene Name** | **Present in  Muzzey's table^(1)^?** | **Amino Acid Impacted** |
| --- | --- | --- | --- | --- | --- | --- | --- |
| **1** | Ca22chr1A | 296097 | C1_01500W | orf19.3336 |  | yes | R/* |
| **2** | Ca22chr1A | 744053 | C1_03540C | orf19.3057 |  | yes | Q/* |
| **3** | Ca22chr1A | 821187 | C1_03880C | orf19.4461 |  | yes | C/* |
| **4** | Ca22chr1A | 854313 | C1_04060W | orf19.4482 | *IFI3* | yes | W/* |
| **5** | Ca22chr1A | 881789 | C1_04250C | orf19.1060 |  | yes | L/* |
| **6** | Ca22chr1A | 1131425 | C1_05380C | orf19.427 |  | yes | Q/* |
| **7** | Ca22chr1A | 1255338 | C1_05970W | orf19.2449 |  | yes | E/* |
| **8** | Ca22chr1A | 1255367 | C1_05970W | orf19.2449 |  | yes | Y/* |
| **9** | Ca22chr1A | 1286182 | C1_06140C | orf19.2431 |  | yes | Y/* |
| **10** | Ca22chr1A | 1286232 | C1_06140C | orf19.2431 |  | yes | R/* |
| **11** | Ca22chr1A | 1711007 | C1_07860W | orf19.5057 |  | yes | S/* |
| **12** | Ca22chr1A | 1760072 | C1_08070W | orf19.5079 | *CDR4* | yes | Y/* |
| **13** | Ca22chr1A | 1882812 | C1_08600C | orf19.407 | *GCD6* | yes | W/* |
| **14** | Ca22chr1A | 1972370 | C1_09060C | orf19.4763 |  | yes | L/* |
| **15** | Ca22chr1A | 1986384 | C1_09100W | orf19.4767 | *ZCF28* | yes | S/* |
| **16** | Ca22chr1A | 2175131 | C1_09960W | orf19.4862.2 | *PET100* | yes | L/* |
| **17** | Ca22chr1A | 2461704 | C1_11200W | orf19.2296 |  | yes | Q/* |
| **18** | Ca22chr1A | 2466747 | C1_11210C | orf19.681 | *HAP43* | yes | Y/* |
| **19** | Ca22chr1A | 2801839 | C1_12860C | orf19.4916 |  | yes | Q/* |
| **20** | Ca22chr1A | 2808532 | C1_12900W | orf19.4921 |  | yes | Q/* |
| **21** | Ca22chr1A | 2915254 | C1_13370W | orf19.4963 |  | yes | L/* |
| **22** | Ca22chr1A | 2959172 | C1_13490C | orf19.4981 |  | yes | Q/* |
| **23** | Ca22chr2A | 466036 | C2_02350C | orf19.1555 | *SAC3* | yes | S/* |
| **24** | Ca22chr2A | 615045 | C2_03050W | orf19.5796 | *SHE9* | yes | L/* |
| **25** | Ca22chr2A | 693284 | C2_03340W | orf19.894 |  | yes | Y/* |
| **26** | Ca22chr2A | 928706 | C2_04430W | orf19.4509 |  | yes | Y/* |
| **27** | Ca22chr2A | 929603 | C2_04440W | orf19.4508 |  | yes | L/* |
| **28** | Ca22chr2A | 980303 | C2_04670W | orf19.149 |  | yes | Y/* |
| **29** | Ca22chr2A | 1388153 | C2_06760C | orf19.2227 |  | yes | L/* |
| **30** | Ca22chr2A | 1585971 | C2_07750W | orf19.2204.2 | *RHO2* | yes | W/* |
| **31** | Ca22chr2A | 1779322 | C2_08750W | orf19.3600 |  | yes | E/* |
| **32** | Ca22chr2A | 2054077 | C2_10030C | orf19.1779 | *MP65* | yes | E/* |
| **33** | Ca22chr2A | 2065445 | C2_10070W | orf19.1774 |  | yes | Q/* |
| **34** | Ca22chr3A | 336960 | C3_01550C | orf19.1690 | *TOS1* | yes | Y/* |
| **35** | Ca22chr3A | 445420 | C3_02020W | orf19.1646 |  | yes | R/* |
| **36** | Ca22chr3A | 530036 | C3_02420C | orf19.230 |  | yes | L/* |
| **37** | Ca22chr4A | 166194 | C4_00910C | orf19.4703 |  | yes | R/* |
| **38** | Ca22chr4A | 166523 | C4_00910C | orf19.4703 |  | yes | W/* |
| **39** | Ca22chr4A | 235553 | C4_01170C | orf19.4673 | *BMT9* | yes | Q/* |
| **40** | Ca22chr4A | 531045 | C4_02560C | orf19.2745 | *UME7* | yes | L/* |
| **41** | Ca22chr4A | 659155 | C4_03130W | orf19.2677 | *GPI16* | yes | R/* |
| **42** | Ca22chr4A | 792650 | C4_03720C | orf19.1306 |  | yes | S/* |
| **43** | Ca22chr4A | 796679 | C4_03750C | orf19.1303 | *MRF2* | yes | R/* |
| **44** | Ca22chr4A | 833072 | C4_03910W | orf19.1596 | *FGR28* | yes | L/* |
| **45** | Ca22chr4A | 833643 | C4_03910W | orf19.1596 | *FGR28* | yes | Y/* |
| **46** | Ca22chr4A | 874950 | C4_04080C | orf19.5302 | *PGA31* | yes | Y/* |
| **47** | Ca22chr4A | 1104475 | C4_05120C | orf19.741 |  | yes | W/* |
| **48** | Ca22chr4A | 1262912 | C4_05720W | orf19.1257 |  | yes | W/* |
| **49** | Ca22chr4A | 1536852 | C4_06900W | orf19.3121 | *GST1* | yes | K/* |
| **50** | Ca22chr4A | 1544443 | C4_06950W | orf19.3114 |  | yes | W/* |
| **51** | Ca22chr4A | 1580763 | C4_07130W | orf19.3091 |  | yes | E/* |
| **52** | Ca22chr5A | 296955 | C5_01330W | orf19.1935 |  | yes | W/* |
| **53** | Ca22chr5A | 484392 | C5_02180C | orf19.4225 | *LEU3* | yes | Q/* |
| **54** | Ca22chr5A | 524833 | C5_02360C | orf19.4244 |  | yes | W/* |
| **55** | Ca22chr5A | 691310 | C5_03120W | orf19.4341 |  | yes | Q/* |
| **56** | Ca22chr5A | 747018 | C5_03300C | orf19.2650 |  | yes | S/* |
| **57** | Ca22chr6A | 24866 | C6_00230W | orf19.1181 |  | yes | W/* |
| **58** | Ca22chr6A | 801191 | C6_03710W | orf19.5742 | *ALS9* | yes | S/* |
| **59** | Ca22chr7A | 692267 | C7_03190C | orf19.5137 |  | yes | Y/* |
| **60** | Ca22chr7A | 746359 | C7_03400C | orf19.1335 | *MTR4* | yes | K/* |
| **61** | Ca22chrRA | 419533 | CR_01870C | orf19.2585 |  | yes | Y/* |
| **62** | Ca22chrRA | 677885 | CR_02930W | orf19.2850 |  | yes | L/* |
| **63** | Ca22chrRA | 803707 | CR_03620C | orf19.4380.1 |  | yes | K/* |
| **64** | Ca22chrRA | 922066 | CR_04110W | orf19.494 |  | yes | C/* |
| **65** | Ca22chrRA | 1018552 | CR_04670C | orf19.1737 |  | yes | Y/* |
| **66** | Ca22chrRA | 1136971 | CR_05270W | orf19.1008 |  | yes | W/* |
| **67** | Ca22chrRA | 1137061 | CR_05270W | orf19.1008 |  | yes | W/* |
| **68** | Ca22chrRA | 1603863 | CR_07380C | orf19.6131 | *KSR1* | yes | R/* |
| **69** | Ca22chrRA | 1734063 | CR_07930C | orf19.605 |  | yes | E/* |
| **70** | Ca22chrRA | 1782353 | CR_08200C | orf19.6382 |  | yes | Q/* |

Grey’s represent multiple pre-mature STOP codons within the same ORF

Confirmed RLAs in red and recessive deleterious allele in yellow

(1) Muzzey D, Schwartz K, Weissman JS, Sherlock G. 2013. Assembly of a phased diploid *Candida albicans* genome facilitates allele-specific measurements and provides a simple model for repeat and indel structure. Genome Biol 14:R97. <https://doi.org/10.1186/gb-2013-14-9-r97>.
