# Supplementary material for: Identification of Recessive Lethal Alleles in the Diploid Genome of a Candida albicans Laboratory Strain Unveils a Potential Role of Repetitive Sequences in Buffering Their Deleterious Impact
Source: mSphere. 2019 Feb 13;4(1):e00709-18. doi: 10.1128/mSphere.00709-18 (PMC6374597; doi:10.1128/mSphere.00709-18)
Supplement: TABLE S2 [file mSphere.00709-18-st002.docx]

**Table S2: Plasmids used in this study**

| **Plasmid** | **Usage** | **Reference** |
| --- | --- | --- |
| pCRBluntIITDH3-GFP-ARG4 | Yeast Fluorescent Cassette | (1) |
| pCRBluntII-TDH3p-BFP-CdHIS1 | Yeast Fluorescent Cassette | (1) |
| pFA-URA3-ISceI_CDR3/TG(GCC)2 | I-*Sce*I TS downstream *mrs-*7b | (2) |
| pCRII::HSP90 | I-*SceI* TS upstream *mrs*-7b | This study |
| pCRII::CUP9 | I-*SceI* TS upstream *mrs*-7b | This study |
| pFA-URA3-ISceI_CDR3/CUP9 | I-*SceI* TS upstream *mrs*-7b | This study |
| pFA-URA3-ISceI_HSP90/CUP9 | I-*SceI* TS upstream *mrs*-7b | This study |
| pDONR207 | *MTR4* recomplementation | (3) |
| pDONR207-MTR4 | *MTR4* recomplementation | This study |
| CIp-pTDH3-GTW-LEU2 | *MTR4* recomplementation | (2) |
| pCRII-IMH3r | *MTR4* recomplementation | This study |
| CIp-pTDH3-GTW-IMH3r | *MTR4* recomplementation | This study |
| CIp-pTDH3-MTR4-IMH3r | *MTR4* recomplementation | This study |

**References:**

1. Loll-Krippleber R, Feri A, Nguyen M, Maufrais C, Yansouni J, d’Enfert C, Legrand M. 2015. A FACS-Optimized Screen Identifies Regulators of Genome Stability in *Candida albicans*. Eukaryot Cell 14:311–322. <https://doi.org/10.1128/EC.00286-14>.

2. Feri A, Loll-Krippleber R, Commere P-H, Maufrais C, Sertour N, Schwartz K, Sherlock G, Bougnoux M-E, D’Enfert C, Legrand M. 2016. Analysis of repair mechanisms following an induced double strand break uncovers recessive deleterious alleles in the *Candida albicans* diploid genome. mBio 7:e1109-16. <https://doi.org/10.1128/mBio.01109-16>.

3. Morschhäuser J, Staib P, Köhler G. 2005. Targeted Gene Deletion in *Candida albicans* Wild-Type Strains by MPA^R^ Flipping, p. 35–44. *In* Ernst EJ, Rogers, PD (ed.), Antifungal Agents: Methods and Protocols. Humana Press, Totowa, NJ.
