# Supplementary material for: Identification of Recessive Lethal Alleles in the Diploid Genome of a Candida albicans Laboratory Strain Unveils a Potential Role of Repetitive Sequences in Buffering Their Deleterious Impact
Source: mSphere. 2019 Feb 13;4(1):e00709-18. doi: 10.1128/mSphere.00709-18 (PMC6374597; doi:10.1128/mSphere.00709-18)
Supplement: TEXT S1 [file mSphere.00709-18-s0001.docx]

**Supplemental methods:**

**Plasmid constructions**

A plasmid carrying a replacement cassette to introduce the I-*Sce*I target sequence (TS) on the right arm of Chr7, near the centromere, was constructed to be integrated in the 8 kb gene-free region between *HSP90* and *CUP9***.** This plasmid carries the *URA3* auxotrophic marker and the I*-Sce*I TS (5’-ATTACCCTGTTATCCCTA-3’) flanked by two 1 kb genomic regions homologous to the Chr7 integration locus in proximity of the centromere (Fig. S1). Both Chr4 homology regions, from the pFA-*URA3*-I*Sce*I-TS-*CDR3*/TG(GCC)2 plasmid (1), were replaced by Chr7 homology regions. These two homology portions are composed of sequences from the Chr7 intergenic region located between *HSP90* and *CUP9* from SC5314 gDNA. The first region at position 445,752-446,549, closer to *HSP90*, was amplified with primers 18 and 19 (Table S2), which possess *Sac*II and *Hpa*I restriction site tails, respectively. The second amplified region (primers 16 and 17) at position 446,703-447,696, closer to *CUP9*, carries tails that contain the I-*Sce*I TS-*Pst*I and *Hind*III restriction sites, respectively. These PCR fragments were cloned into the TOPO-TA pCRII plasmid yielding plasmids pCRII::*HSP90* and pCRII::*CUP9*. pFA-*URA3*-I*Sce*I-TS-*CDR3*/TG(GCC)2 was digested with *Pst*I and *Hind*III to replace one of the Chr4 regions with the *Pst*I,*Hind*III-digested fragment from pCRII::*CUP9*. The resulting plasmid pFA-*URA3*-I*Sce*I-TS-*CDR3*/*CUP9* and pCRII::*HSP90* were both digested with *Hpa*I and *Sca*II to replace the second Chr4 region by the second Chr7 region, resulting in pFA-*URA3*-I*Sce*I-TS-*HSP90/CUP9*.

For *MTR4* complementation at the *RPS1* locus, the CIp10-P*_TDH3_*-*MTR4*-*IMH3* plasmid containing the wildtype allele of *MTR4* expressed under the control of the constitutive promoter P*_TDH3_* and associated to the *IMH3* gene conferring resistance to MPA was constructed. To this aim, the *IMH3* gene was amplified by PCR (Expand^TM^ Long range dNTPack, Roche) from *pSF*II (2) and cloned in the TOPO^®^-TA pCRII vector (ThermoFisher Scientific). The *IMH3* fragment was then recovered from the resulting plasmid by *Sac*II digestion and cloned in the *Sac*II-linearized CIp10-P*_TDH3_*-GTW-*LEU2* (1) giving rise to CIp10-P*_TDH3_*-GTW-*IMH3*. The ORF coding for *MTR4* was amplified by PCR using primers 25 and 26 (Table S2) containing aatP1 and attP2 sites for Gateway™-recombinational cloning and transferred in pDONR207 using the BP clonase™ as previously described (3). Several recombined plasmids were sequenced to confirm the cloned haplotype. pDONR207::*MTR4A* containing *MTR4* haplotype A was then used in combination with CIp10-P*_TDH3_*-GTW-*IMH3* and LR clonase™ (Invitrogen) to yield CIp10-P*_TDH3_*-*MTR4*-*IMH3.*

***C. albicans* strain constructions**

Supplementary:

The parental strain CEC4591 (Table 1) was sequentially transformed by heat shock according to the Lithium Acetate/PEG protocol (4), in order to obtain the final constructions represented in Fig. 1A and Supplemental Fig. S1. CEC4591 is derived from the common laboratory strain SN76 and carries the I-*Sce*I gene under the control of the inducible P*_TET_* promoter (5), as well as pNIMX (6) coding for the transactivator necessary for the proper activation of the P*_TET_* promoter in presence of tetracycline derivatives. The strategy was to introduce (i) the BFP/GFP LOH reporter system on the right arm of Chr7 near the telomeres and (ii) the I-*Sce*I TS on the right arm of Chr7 near the centromeres of each Chr7 homolog. CEC4591 was first transformed with the P*_TDH3_*-*GFP*-*ARG4* (7) cassette that was designed to integrate by homologous recombination on the right arm of Chr7 at position 911,043-911,337, in a roughly 5 kb intergenic region (Fig.1A and Fig. S1). To do so, 120 bp primers were used, which are composed of 100 bp tails possessing the complementary sequence of the Chr7 integration locus and 20 bp complementary to the P*_TDH3_*-*GFP*-*ARG4* cassette. Thus, primers 1 and 2 (Table S2) were used to amplify by PCR the P*_TDH3_*-*GFP*-*ARG4* integration cassette from plasmid pCRBluntII-P*_TDH3_*-*GFP*-*ARG4*. The cassette was amplified in a total PCR reaction volume of 500 μL, precipitated overnight in 50 μL of sodium acetate and 1 mL of 100% ethanol, pelleted by centrifugation at maximum speed for 30 min at 4°C and suspended in 25 μL of 1X TE Buffer for transformation in *C. albicans*. Transformants were selected on SC-Arg agar medium and validated by PCR to verify both boundaries of the integration, giving rise to CEC4679. Similarly, CEC4679 was subsequently transformed with the P*_TDH3_*-*BFP*-*HIS1* cassette (7) that was designed to integrate at the same allelic locus as the P*_TDH3_*-*GFP*-*ARG4* cassette hence resulting in the complete artificial heterozygous BFP/GFP locus. The P*_TDH3_*-*BFP*-*HIS1* integration cassette was amplified from plasmid pCRBluntII-P*_TDH3_*-*BFP*-Cd*HIS*1. Transformants were selected on SD+Uri plates and junctions were validated by PCR (Table S2). This insured that transformants possess simultaneously the P*_TDH3_*-*GFP*-*ARG4* and the P*_TDH3_*-*BFP*-*HIS1* cassettes, each one localized on a different chromosomal homologue. The resulting strain was CEC4685.

References:

1. Feri A, Loll-Krippleber R, Commere P-H, Maufrais C, Sertour N, Schwartz K, Sherlock G, Bougnoux M-E, D’Enfert C, Legrand M. 2016. Analysis of repair mechanisms following an induced double strand break uncovers recessive deleterious alleles in the *Candida albicans* diploid genome. mBio 7:e1109-16. <https://doi.org/10.1128/mBio.01109-16>.

2. Morschhäuser J, Staib P, Köhler G. 2005. Targeted Gene Deletion in *Candida albicans* Wild-Type Strains by MPA^R^ Flipping, p. 35–44. *In* Ernst EJ, Rogers, PD (ed.), Antifungal Agents: Methods and Protocols. Humana Press, Totowa, NJ.

3. Legrand M, Bachellier-Bassi S, Lee KK, Chaudhari Y, Tournu H, Arbogast L, Boyer H, Chauvel M, Cabral V, Maufrais C, Nesseir A, Maslanka I, Permal E, Rossignol T, Walker LA, Zeidler U, Znaidi S, Schoeters F, Majgier C, Julien RA, Ma L, Tichit M, Bouchier C, Dijck P V, Munro CA, D’Enfert C. 2018. Generating genomic platforms to study *Candida albicans* pathogenesis. Nucleic Acids Res 46:6935-6949. <https://doi.org/10.1093/nar/gky594>.

4. Gola S, Martin R, Walther A, Dünkler A, Wendland J. 2003. New modules for PCR-based gene targeting in *Candida albicans*: rapid and efficient gene targeting using 100 bp of flanking homology region. Yeast 20:1339–1347.

5. Park Y-N, Morschhäuser J. 2005. Tetracycline-inducible gene expression and gene deletion in *Candida albicans*. Eukaryot Cell 4:1328–1342.

6. Chauvel M, Nesseir A, Cabral V, Znaidi S, Goyard S, Bachellier-Bassi S, Firon A, Legrand M, Diogo D, Naulleau C, Rossignol T, d'Enfert C. 2012. A Versatile Overexpression Strategy in the Pathogenic Yeast *Candida albicans*: Identification of Regulators of Morphogenesis and Fitness. PLoS One 7:e45912. <https://doi.org/10.1371/journal.pone.0045912>.

7. Loll-Krippleber R, Feri A, Nguyen M, Maufrais C, Yansouni J, d’Enfert C, Legrand M. 2015. A FACS-Optimized Screen Identifies Regulators of Genome Stability in *Candida albicans*. Eukaryot Cell 14:311–322. <https://doi.org/10.1128/EC.00286-14>.
