# Supplementary material for: Identification of Recessive Lethal Alleles in the Diploid Genome of a Candida albicans Laboratory Strain Unveils a Potential Role of Repetitive Sequences in Buffering Their Deleterious Impact
Source: mSphere. 2019 Feb 13;4(1):e00709-18. doi: 10.1128/mSphere.00709-18 (PMC6374597; doi:10.1128/mSphere.00709-18)
Supplement: TABLE S3 [file mSphere.00709-18-st003.docx]

**Table S3:** **Primers used in this study.**

| **Primer** | **Name** | **Sequence** | **Usage** |
| --- | --- | --- | --- |
| **1** | K7_yFP_Ch7Right_Fwd | CACCGATAGGAATCTGGTCCAGAAGATTGGTTGTCATGTATTTTGGTCTATAAGAATATGAGGTGACAACTTGCATTGGTGTGTAGTAGATTTCTGGTTGCAGGAAACAGCTATGACC | Integration of the BFP/GFP LOH reporter system |
| **2** | K7_yFP_Ch7Right_Rev | TTTTCCTTTAGGCAAGTTCGCCTTTAGAGAAGATTCCATTTATCAGAGGTTGAAACTAAAAGAGGCGAAGAGCTACTTTATTGTCACCGAGCATGCTTGGGTTTTCCCAGTCACGACG | Integration of the BFP/GFP LOH reporter system |
| **3** | Ch7_Right_YFP_Integration_F | CCATTTGACAAGTTTAGAGGAGAC | Integration of the BFP/GFP LOH reporter system |
| **4** | Ch7_Right_YFP_Integration_R | GTTTACAAACCCTGGGTCTC | Integration of the BFP/GFP LOH reporter system |
| **5** | CaTDH3-R-BsiWI | AGAGAGCGTACGTGTAAAGTTTGTTGATGTTAA | Integration of the BFP/GFP LOH reporter system |
| **6** | S Arg1 | GCTACCGATATGAGAATTTTCGTTCG | Integration of the BFP/GFP LOH reporter system |
| **7** | CdHIS1_detectF | AATGCTGCAGCTTATTGAGCGGTG | Integration of the BFP/GFP LOH reporter system |
| **8** | CdHIS1_detectR | TGCCCTTCTACCTGGAGTAATGGT | Integration of the BFP/GFP LOH reporter system |
| **9** | K7_URA3_ISceI_F | GAAATTGGTTTGTTCATTTAAAAAATGGGTTATGCAACACTGCCCAGTTCTAGCTTGTTGTAATTATTGTAGTGGTCC**ATTACCCTGTTATCCCTA**GCTCGTTTAAACTAGAAGGACCAC | Integration of I-SceI TS downstream of the mrs-7b |
| **10** | K7_URA3_ISceI_R | TTCCTCTCGACACTGCCCAATAAAATTGTTCTGCCTCTGTTGATAAGGCGGTACAAATTTAGTTGAGCCCATTACTTGGTTGTTTTGATCCTTTACGCATCGACGGATCCGGATGGTATA | Integration of I-SceI TS downstream of the mrs-7b |
| **11** | K7_URA3_ISceI_verif_F | ACCACCGGTAGCAACATTCT | Integration of I-SceI TS downstream of the mrs-7b |
| **12** | K7_URA3_ISceI_verif_R | ATGGTTGTGCACGTGATCAA | Integration of I-SceI TS downstream of the mrs-7b |
| **13** | URA3verfor | GAGGATTGTTTGGTAAAGGAAGAG | Integration of I-SceI TS downstream of the mrs-7b |
| **14** | C7_02030w_DWN_F | GGGAAAGTTAACCCTTGGCTGTAATGAGCCTTG | Integration of I-SceI TS upstream of the mrs-7b |
| **15** | C7_02030w_DWN_R | AAAGGGCCGCGGGTGGATGGGTAATACTTGCCTG | Integration of I-SceI TS upstream of the mrs-7b |
| **16** | C7_02040c_UP_F | GGGAAACTGCAG**ATTACCCTGTTATCCCTA**CAGTGACTAACGTTTCCAATGG | Integration of I-SceI TS upstream of the mrs-7b |
| **17** | C7_02040c_UP_R | AAAGGGAAGCTTGCTATTGCATACGGAGATATCTC | Integration of I-SceI TS upstream of the mrs-7b |
| **18** | Verif_C7_02030c_F | CCTGGTAGAACATGAGCTCT | Integration of I-SceI TS upstream of the mrs-7b |
| **19** | Verif_C7_02040w_R | GGAGTGTCAATTACGGATTG | Integration of I-SceI TS upstream of the mrs-7b |
| **20** | SNPs_414508_414582_F | AGCACTCGCCAATCGTGATA | SNP-RFLP |
| **21** | SNPs_414508_414582_R | TCATCATCGTCGACGTCATC | SNP-RFLP |
| **22** | SNP444929_F | AGCAGTATCAACGCCAGCAT | SNP-RFLP |
| **23** | SNP744964_F | ACATCGAACCTCTAGGCGTAG | SNP-RFLP |
| **24** | SNP273287_R | TGCTTTGGACACCATGATGC | SNP-RFLP |
| **25** | 61_E06_F | GGGGACAAGTTTGTACAAAAAAGCAGGCTTGATGGATGACTTGTTTGATGTCTTTGATGAA | *MTR4* recomplementation |
| **26** | 61_E06_R | GGGGACCACTTTGTACAAGAAAGCTGGGTCCATATACAATGAAGATACTTGTACAAAATC | *MTR4* recomplementation |
| **27** | MTR4_SNP_TM_F | AGTTCGACTGCCAAGAGAACA | *MTR4* recomplementation |
| **28** | MTR4_SNP_TM_R | CTGGTTCAGCAAGTTCTGGT | *MTR4* recomplementation |
| **29** | MTR4_ORF_Verif1_F | CCTCAAGCAGCTCCACCTAA | *MTR4* recomplementation |
| **30** | MTR4_ORF_Verif2_F | TCTGCTGGTAAAACCGTGGT | *MTR4* recomplementation |
| **31** | MTR4_ORF_Verif3_F | GACGACCCTGCTTCAAGTGA | *MTR4* recomplementation |
| **32** | MTR4_ORF_Verif4_F | AGACGTGGGTTGGATGATCG | *MTR4* recomplementation |
| **33** | MTR4_ORF_Verif5_F | GTACGTAAAGTTATTACTCACCCAGG | *MTR4* recomplementation |
| **34** | MTR4_ORF_Verif6_F | TGAAAGGTAGAGTAGCTGCTGA | *MTR4* recomplementation |
| **35** | CaIMH3r_SacI_F | CCCAAAGAGCTCGTCGACGGTATCGATAAG | *MTR4* recomplementation |
| **36** | CaIMH3r_SacI_R | CCCAAAGAGCTCCTGCAGGAATTCGATGTA | *MTR4* recomplementation |
| **37** | IMH3r_verif_F | AATTCGAAGGGGGCGTTCAT | *MTR4* recomplementation |
| **38** | IMH3r_verif_R | ACCGATAAACCATCTTTCTTAGGGT | *MTR4* recomplementation |

Restriction sites are underlined while the I-*Sce*I target sequence is identified in bold blue font
